# Comparative analyses of transcriptional responses of *Dectes texanus* LeConte (Coleoptera: Cerambycidae) larvae fed on three different host plants and artificial diet

**DOI:** 10.1038/s41598-021-90932-x

**Published:** 2021-06-01

**Authors:** Lina M. Aguirre-Rojas, Erin D. Scully, Harold N. Trick, Kun Yan Zhu, C. Michael Smith

**Affiliations:** 1 Deparment of Botany and Plant Sciences, University of California Riverside, Riverside, CA 92506 USA; 2 Stored Product Insect and Engineering Research Unit, USDA-ARS-CGAHR, Manhattan, KS 66502 USA; 3 Department of Plant Pathology, Kansas State University, Manhattan, KS 66506 USA; 4 Department of Entomology, Kansas State University, Manhattan, KS 66506 USA

**Keywords:** Entomology, Gene expression, RNA sequencing

## Abstract

*Dectes texanus* is an important coleopteran pest of soybeans and cultivated sunflowers in the Midwestern United States that causes yield losses by girdling stems of their host plants. Although sunflower and giant ragweed are primary hosts of *D. texanus*, they began colonizing soybeans approximately 50 years ago and no reliable management method has been established to prevent or reduce losses by this pest. To identify genes putatively involved when feeding soybean, we compared gene expression of *D. texanus* third-instar larvae fed soybean to those fed sunflower, giant ragweed, or artificial diet. *Dectes texanus* larvae differentially expressed 514 unigenes when fed on soybean compared to those fed the other diet treatments. Enrichment analyses of gene ontology terms from up-regulated unigenes in soybean-fed larvae compared to those fed both primary hosts highlighted unigenes involved in oxidoreductase and polygalacturonase activities. Cytochrome P450s, carboxylesterases, major facilitator superfamily transporters, lipocalins, apolipoproteins, glycoside hydrolases 1 and 28, and lytic monooxygenases were among the most commonly up-regulated unigenes in soybean-fed larvae compared to those fed their primary hosts. These results suggest that *D. texanus* larvae differentially expressed unigenes involved in biotransformation of allelochemicals, digestion of plant cell walls and transport of small solutes and lipids when feeding in soybean.

## Introduction

Emergence of arthropod pests in agronomically important crops is often linked to host range expansions^1^. Host range expansions are observed in arthropod herbivores that are already inherently capable of colonizing a broad range of plant species within or different taxonomic families^1–7^. Novel plant-arthropod herbivore associations can be formed between previously geographically separated organisms or organisms that have co-existed in the same landscape^6^. These host range shifts are often attributed to low availability of natural hosts, escape from predation and/or parasitism, availability of more attractive or better quality host plants, availability of hosts that are less chemically defended, and introduction or invasion to new landscapes^8,9^. Plasticity of arthropod gene expression is most likely associated with the initial ability of herbivores to use and feed on novel and multiple host plants^10–12^. In general, immediate genomic changes may not be initially required for an arthropod to use a novel host plant^3^. Over time, gene duplications and selection for novel alleles are associated with changes in digestive physiology and detoxification, allowing arthropods to continue to feed on new or chemically defended host plants^13–15^.

Analyses of transcriptomes of arthropod herbivores fed natural and novel hosts indicate that differences in host plant chemistry have a variety of different impacts on herbivore gene expression depending on the relationship between the plant host and insect species. For example, transcriptional differences in the ways in which specialist and generalist herbivores respond to feeding on their primary or secondary plant hosts have been observed. Two previous studies showed that some generalist species exhibit broader transcriptomic responses to cope with the detrimental effects of plant toxins on metabolism, nutrition and growth when reared on alternate hosts whereas more narrow and attuned transcriptomic responses occur in specialists to detoxify plant toxins from their hosts and reduce activation of stress signals^12,16^. In one study, approximately 10% of the contigs in the larvae of the generalist *Heliothis virescens* Fabricius were differentially expressed when fed on *Arabidopsis thaliana* (L.) Heynhold, a secondary host, compared to larvae of the Brassicaceae specialist *Pieris brassicae* L.*,* in which only 1% their contigs were differentially expressed after feeding on the same plant. In a second study, the specialist herbivore *Manduca sexta* L. fed on its secondary hosts *Datura wrightii* Regel and *Brassica napus* L. differentially expressed 5.2 and 3.6 times more genes compared to insects fed on its primary host *Nicotiana attenuata* Torr. ex. Watson^11^. In contrast, the *Passiflora* L. specialist *Heliconius melpomene* L. fed *Passiflora biflora* Lamarck up-regulated fewer genes compared to those fed its primary host, *Passiflora menispermifolia* Kunth^17^. In addition, transcriptomic changes can become more attuned over time as a population or species adapts to its new host plant^18,19^. For example, larvae of the maize-strain of *Spodoptera frugiperda* Smith exhibited fewer changes in gene expression compared to larvae of the rice-strain after both strains ate maize^20^.

Regardless of the magnitude of the transcriptional changes that accompany a host shift, arthropod herbivores must cope with plant defense compounds and modulate plant defense responses to feed and acquire plant nutrients. Differential expression of herbivore genes involved in digestion; detoxification and inactivation of plant chemical defenses; and maintaining the structural integrity of the peritrophic matrix (PM) are thought to be key factors involved in feeding, survival and adaptation of herbivores to novel host and the ability to feed on multiple host plants^10,11,16,18^. During successful feeding, the need to breakdown low-nutrient plant tissues can trigger expression level changes in genes coding for digestive enzymes such as proteases, protease inhibitors, lipases, glycoside hydrolases, and amylases^10,16,21^. Furthermore, cytochrome P450s (CYPs), carboxylesterases (CarEs), UDP-glucuronosyltransferases(UGTs), glutathione *S*-transferases (GSTs) and ATP-binding cassette transporters (ABCs) are often differentially regulated in herbivores feeding on novel host and non-host plants^11,16,17,19^, and are involved in the disarmament and excretion or sequestration of plant allelochemicals^22,23^. Changes in the expression levels of effectors are also thought to be important in the attenuation or suppression of plant defense compounds induced by feeding damage to plant tissues^20,24,25^. Maintaining the integrity of the PM is also essential in to counter plant anti-nutritive defenses^21^. The PM separates the food bolus from the midgut epithelium, protecting this tissue and the rest of the insect from damage by ingested defensive compounds^26^. Up-regulation of components involved in maintaining the structural integrity of this matrix, such as chitin synthase and peritrophin domain-containing proteins, has been observed in arthropods feeding on primary host and non-host plants^16,17,19,20,27^. Although transcriptional changes in genes coding for digestive, detoxification, and PM-related proteins and enzymes are often observed in insects feeding on non-host plants, functional experiments are needed to evaluate whether the ability to regulate the genes mentioned above facilitates host range expansion in arthropods^10,21^.

*Dectes texanus* LeConte (Coleoptera: Cerambycidae), commonly known as the *Dectes* stem borer, is a native long-horned beetle species of North America^28^ and a pest of soybeans (Fabaceae: *Glycine max* (L.) Merr.) and cultivated sunflower (Asteraceae: *Helianthus annus* L.) in several states of the USA. *Dectes texanus* infestations in soybean and sunflower fields occur every year when larvae damage plant stems by tunneling and girdling^29–32^, causing approximately 5–15% and 10% reduction in soybean and sunflower yields, respectively^33–35^. *Dectes texanus* expanded its host range to soybeans more than 50 years ago when it was first reported as a pest of soybeans in Missouri^36–38^. *Dectes texanus* primary host plant species, *Ambrosia trifida* L. (giant ragweed), *H. annus*, and *Xanthium strumarium* L., are members of the Asteraceae family^37^. Although *Dectes texanus* feeds and completes its development inside soybean stems, using this novel host presents fitness costs for this beetle species. Adult females and males, pupae, and larvae reared on sunflower are significantly longer in size and two times heavier than individuals collected from soybeans^39–41^. Differences in larval size and weight may be related to the nutritional quality or stalk width of the host plants^39–41^. Interestingly, there were no weight differences in overwintering larvae collected from soybean compared to those collected from sunflower^42^. Although detoxification enzymes and gene families important for host plant selection and the ability to use alternative hosts have been identified in other cerambycids^43–45^ and insects^46^, it is unknown what genetic factors enable *D. texanus* to colonize, feed and survive on soybeans. To understand how *D. texanus* uses soybean as a new host, we compared the global transcriptome profiles of larvae fed on soybean to larvae fed on sunflower, giant ragweed or artificial diet. Through this approach, we produced the first de novo transcriptome assembly from *D. texanus* larvae and identified unigenes differentially expressed in larvae fed soybean that could be associated with its success in feeding on a novel host (soybean). Ultimately, this research will help us develop novel management tools to make soybean unsuitable for this pest.

## Results 

### *Dectes texanus* genome size

The mean genome size of *D. texanus* females and males was 466.4 Mb (SE = 1) and 463.2 Mb (SE = 0.7), respectively, for an overall mean size of 464.7 Mb (SE = 0.6), which is smaller than the genome of the Asian long-horned beetle, *Anoplophora glabripennis* Motschulsky (710 Mb)^43^. *Dectes texanus* has the smallest known genome size of any other Cerambycid^47^ measured by flow cytometry and reported in the Animal Genome Size Database^48^ (December 18th, 2020).

### *Sequencing and *de novo* transcriptome assembly*

A total of 355.6 million reads were obtained from the 12 RNA-seq libraries derived from third-instar larvae with the total number of read pairs per sample ranging from 25.7 to 33.8 million reads per library (Supplementary Table S1). Approximately ~ 335.4 million (99%) reads from all samples were retained after removing low quality bases and reads. Approximately 11.8 million high quality reads remained after in silico normalization and were used by Trinity to construct the de novo transcriptome assembly.

The raw *D. texanus* transcriptome assembly yielded 127,878 putative transcripts and 65,979 unigenes with N50 contig lengths of 2387 and 1877 bp, respectively (Supplementary Table S2). A total of 19,791 (15.5%) transcripts had low expression values and low support from read coverage compared to the dominant isoform (see methods), and 66,626 (52.1%) transcripts lacked an open reading frame (ORF) and were removed from the assembly. The final filtered assembly contained 41,461 transcripts and 14,504 unigenes with an N50 length of 3025 and 3195 bp, respectively (Supplementary Table S2). The size of the filtered assembly was 97 Mb based on the sum of the transcript lengths and 33.3 Mb based on the sum of the longest transcript per unigene. *Dectes texanus* transcriptome assembly represented approximately 7% of the genome (33.3 Mb/466 Mb). Transcripts with predicted complete, 5′ partial and 3′ partial ORFs represented 73.8, 7.9, and 12.5% of the filtered assembly, respectively. Unigenes with predicted complete, 5′ partial and 3′ partial ORFs represented 68.8, 10.9, and 9.8% of the filtered assembly, respectively (Supplementary Table S2).

Though ~ 93.6% of the protein coding unigenes matched sequences derived from insects, 5 unigenes (0.03%) were derived from plants, 11 unigenes (0.08%) were derived from bacteria, 4 unigenes (0.03%) were derived from fungi, and 26 unigenes (0.2%) were derived from viruses. These non-insect unigenes were removed from the final assembly. Approximately 86% of the *D. texanus* protein coding unigenes matched sequences derived from the order Coleoptera, where 54.1% of the unigenes had highest scoring matches to sequences derived from the family Cerambycidae (Supplementary Fig. S1). *Dectes texanus* protein coding unigenes matching sequences from the orders Lepidoptera, Hymenoptera, Hemiptera and Diptera were 0.3%, 0.98%, 0.28% and 0.14%, respectively, and 5.8% matched to insects, but could not be assigned to an order using MEGAN.

The filtered transcriptome was functionally annotated using BLASTp and HMMER searches against the Uniprot/SwissProt and PFAM-A databases, respectively, where 10,307 (71.1%) and 10,471 (72.2%) of the unigenes had at least one BLASTp match to an Uniprot/SwissProt protein and a Pfam-A domain, respectively (Supplementary Table S3). In addition, at least one gene ontology (GO) term and/or KEGG (Kyoto Encyclopedia of Genes and Genomes^49–51^) orthology (KO) term was predicted for 6,772 (46.7%) and 5,362 (37%) of the unigenes, respectively (Supplementary Table S3).

Benchmarking Universal Single-Copy Ortholog (BUSCO) analysis was performed at the unigene level, and the KEGG pathway representation was compared to *A. glabripennis* to gauge the completeness of the *D. texanus* transcriptome assembly in terms of recovered gene space. The analysis led to the recovery of 1,570 (94.6%) complete BUSCOs of the Insecta ODB9 linage gene set with 93.8%, 0.8%, and 1.6% complete single-copy, duplicated, and fragmented BUSCOs, respectively, indicating that the majority of the conserved insect genes were captured in the assembly. The low number of duplicated BUSCOs suggested that the majority of the transcripts derived from the same locus had been successfully collapsed to the unigene level by Trinity. Providing further support to the high quality of the *D. texanus* transcriptome assembly, the number of *D. texanus* unigenes assigned to core KEGG metabolic pathways that are expected to be represented in the majority of insect taxa were similar to those previously annotated in the *A. glabripennis* genome (Supplementary Fig. S2). The number of unique KO terms found in the *D. texanus* transcriptome were also similar to those represented in the genomes of two other beetle species, including *Tribolium castaneum* Herbst and *Dendroctonus ponderosae* Hopkins (Supplementary Table S4). These data indicated that several conserved metabolic pathways were well represented in the transcriptome assembly, suggesting that the *D. texanus* transcriptome contains a comprehensive representation of the majority of KEGG metabolic pathway genes.

### Glycoside hydrolases involved in plant cell wall degradation

Cerambycids rely on sugars from plant cell wall polysaccharides, including cellulose, pectin, and hemicelluloses, as the main carbon sources for their growth and development^52^. Glycoside hydrolases (GHs) are important enzymes in stem- and wood-boring insects that facilitate digestion of major classes of plant cell wall polysaccharides into absorbable monosacharides^45,53^. Also, GHs are relevant for the biotransformation of plant defensive compounds that contain glycosidic linkages^54,55^. A brief descriptive summary of these families in the *D. texanus* assembly are described herein since they may be relevant in the use of soybean as a host plant.

Twenty-two different glycoside hydrolase families, spanning 120 unigenes, were identified from the *D. texanus* transcriptome (Supplementary Fig. S3). The number of GH families identified is 1.6 times higher than those previously identified in the *A. glabripennis*-midgut de novo transcriptome^56^, most likely due the fact that the *D. texanus* transcriptome was assembled with RNA-seq data from whole larvae fed three different plant species while the *A. glabripennis* transcriptome was constructed using guts from larvae fed only on one tree species. However, the number of GH families identified in the *D. texanus* transcriptome was ~ 4% lower than the number of GH families encoded in the *A. glabripennis* genome^43^.

Overall, GH1 was the most highly represented family in the *D. texanus* transcriptome associated with plant cell wall degradation (Supplementary Fig. S3a and S4); however, the number of unigenes identified in this family is 35% and 39% less than those previously reported in the *A. glabripennis* transcriptome^56^ and genome^43^. Unigenes coding for GH1 enzymes in *D. texanus* had highest scoring BLASTp matches to proteins annotated as myrosinase 1, myrosinase 1-like, lactase-phlorizin hydrolase and lactase from the Uniprot database. Interestingly, the GH5 and GH48 families had two and five more *D. texanus* unigenes identified relative to those annotated in the *A. glabripennis* genome (Supplementary Fig. S3a), respectively. Members of the GH5 family had highest scoring BLASTp matches to proteins annotated as endoglucanase Z and endoglucanase 5A, while members of the GH48 family had matches to exoglucanase B. Genome sequencing will be required to validate whether these unigenes are coded by separate loci/genes in *D. texanus* or whether they represent allelic variants from the same locus. Other unigenes coding for endoglucanases were assigned to the GH9 and GH45 families (Supplementary Fig. S3a) and most likely confer the ability to digest two of the most prominent plant secondary cell wall polysaccharides (cellulose and hemicellulose) in *D. texanus*, as previously indicated in *A. glabripennis* through *in-vitro* functional characterization^43^.

The GH28 and GH38 families had 12 and 10 *D. texanus* unigenes with high scoring BLASTp matches to poly- or endo-galacturonases, and α-mannosidases, respectively. Putatively, these enzymes are most likely important for *D. texanus* in the hydrolysis of polygalacturonan (pectin) and hemicellulose (glycan heteropolymers) when digesting stem and petiole pith, respectively^57^. The GH18 family was the second most abundant GH family in the *D. texanus* transcriptome and had 18 unigenes with high scoring BLASTp matches to chitinases (Supplementary Fig. S4). Insect chitinases belong to the GH18 family^58^.

### Enzymes involved in biotransformation of plant allelochemicals

UGTs, GSTs, CarEs, CYPs and ABC transporters are also key enzymes involved in the biotransformation of plant allelochemicals and confer the ability to use a broad range of plant hosts in many insect species^59^. The most prominent classes of biotransformation enzymes identified in the *D. texanus* transcriptome were CYPs (84 unigenes), followed by CarEs (52 unigenes), UGTs (41 unigenes), and GSTs (26 unigenes) (Supplementary Fig. S3b). The total number of unigenes coding for each of these classes was lower than those reported in the *A. glabripennis* genome^43^ and in the transcriptomes of the cerambycids *Monochamus alternatus* Hope^60^ and *Batocera horsfieldi* Hope^61^.

The four clades of CYPs typically found in insect genomes and transcriptomes^62^ were represented in the *D. texanus* transcriptome, and family members of clades 3 and 4 were the most abundant among the unigenes coding for CYPs (Supplementary Fig. S5). Within clade CYP3, 34.5% of the CYP unigenes were assigned to the CYP6 family, and 15.5% were assigned to the CYP9 family. Within clade CYP4, 31% were assigned to the CYP4 family. In contrast, only nine unigenes coding for mitochondrial CYP enzymes spanning six families were identified in the *D. texanus* assembly. Among these unigenes, those coding for enzymes belonging to the CYP49 family were the most frequent. The CYP4, 6, and 9 families include many enzymes associated with insect-plant interactions in other insect taxa^23^, and known members of these families are important in metabolizing a broad spectrum of plant compounds^11^.

The CarE clades found in other coleopterans^63–65^ were represented in the *D. texanus* transcriptome, and each clade had at least one unigene with a complete ORF, except for clade H (Supplementary Fig. S6). Two putative unigenes coding for acetylcholinesterases (Clade J) were found in the *D. texanus* transcriptome, consistent with the number reported in other coleopterans, such as *A. glabripennis*^43^, *Leptinotarsa decemlineata* Say^64^ and *T. castaneum*^66^. Clades A (xenobiotic metabolizing enzymes) and E (β- and pheromone esterases) contained the most *D. texanus* CarE unigenes with complete ORFs, respectively. These two clades were also the most abundant in other cerambycids including the transcriptomes of *Rhaphuma horsfieldi* White and *Xylotrechus quadripes* Chevrolat^65^ and the *A. glabripennis* genome^43,65^*.* For example, an expansion of clade E CarEs was identified in the genome of *A. glabripennis,* and 13 of them were highly induced in larvae fed sugar maple, *Acer saccharum,* compared to those fed artificial diet^43^.

Phylogenetic analyses showed that unigenes from *D. texanus* coding for complete UGT ORFs were classified into 10 different families where families 321, 324, and 411 contained the most unigenes (Supplementary Fig. S7)*.* A member of the UGT50 family was also identified in the *D. texanus* transcriptome. As in previous studies, we detected (1) gene expansions in family 324, which have been observed in other coleopterans and are unique to this order^67^, (2) gene expansions in families 321 as has been observed in other cerambycids^43,67,68^, and (3) the occurrence of multiple unigenes coding for UGTs belonging to families 411 and 412, which are thus far unique to the family Cerambycidae^43,68^. UGT411 and 412 were formerly known as families UGT352 and 353 in *A. glabripennis* and other Cerambycids, respectively^43,65^. These UGT families in Cerambycidae were re-named to distinguish them from those in Hemiptera (New names approved by the UGT nomenclature committee on February 2, 2021). Contribution of UGTs in herbivores to host plant adaptation has been studied using gene expression and silencing experiments, and protein-substrate bioassays in other arthropods. For example, RNA interference-mediated knock-down of UGT330A3, 344D5, 348A3, and 349A3 in *Myzus persicae nicotianae* Blackman increased mortality when eating tobacco, and UGT352A1, 352B1, and 354A1 were up-regulated on *Bemisia tabaci* Gennadius fed cabbage^69,70^. Also, recombinant *Tetranychus urticae* Koch UGT202A and UGT204B and *Helicoverpa armiguera* Hübner UGT41B3 and UGT40D1 were capable of glycosylating capsaicin, multiple flavonoids, and gossypol *in vitro* and are likely involved in detoxifying these allelochemicals. ^71,72^. It has been suggested that UGT411 expansion (formerly known as UGT352) is associated with wide host range and adaptation in *A. glabripennis*^43^*.*

Six cytosolic GST classes were represented in the *D. texanus* transcriptome (Supplementary Fig. S8) where the epsilon class contained the most *D. texanus* GSTs with complete ORFs, followed by the sigma and delta classes. The omega, zeta, and theta classes contained at least one unigene with a complete ORF. Host plant feeding may be related to the composition and expression of GSTs in herbivorous insects where generalists may have expanded copy numbers of GSTs to detoxify plant compounds compared to specialized insects^73^. Overexpression of epsilon and sigma GSTs have been associated with adaptation and detoxification of plant allelochemicals in lepidopterans and coleopterans^74–76^, and expansion of epsilon GSTs may confer resistance to insecticides in *T. castaneum*^73,77^.

All ABC families reported in *T. castaneum*^78^ were represented in the *D. texanus* transcriptome (Supplementary Fig. S9). The most abundant ABC families with complete protein coding sequences were families C (drug conjugate transporter) and G (eye pigment precursor transporter). Families B, D, E, F, and H contained similar number of unigenes to those found in the *A. glabripennis* and *T. castaneum* genomes^43,78^. The interactions between soybean substrates and *D. texanus* CYP, CarE, GST, UGT, ABC unigenes are unknown. Functional characterization of these protein families is needed to understand their importance in host plant adaptation and expansion in *D. texanus*.

## Differentially expressed unigenes in soybean-fed larvae

The average overall alignment rate for each library was 66.8% when it was mapped against the filtered transcriptome assembly containing only transcripts that coded for proteins. The three replicates within soybean, sunflower, and artificial diet-fed larvae were highly correlated (R^2^ > 0.5) with one another, based on global expression profiles and Pearson correlations (Supplementary Fig. S10 and S11). However, the three replicates from larvae fed giant ragweed were not as strongly correlated (R^2^ < 0.3) with one another and the expression profiles of these replicates were more variable (Supplementary Fig. S10). This variability may be attributed to environmental effects (temperature, availability of soil nutrients), plant age differences, or the length of time it took to dissect the stems.

**Figure 1:**
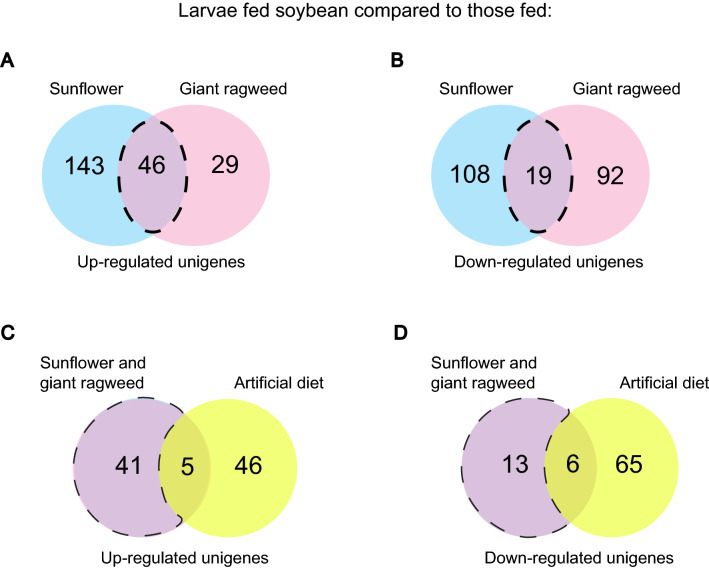
Number of differentially expressed unigenes in *Dectes texanus* larvae fed soybean compared to those fed other diet treatments (Fold change >  ± 1.5, False Discovery Rate < 0.05). (**A**) up-regulated and (**B**) down-regulated unigenes compared to larvae fed sunflower or giant ragweed; (**C**) up-regulated and (**D**) down-regulated unigenes compared to larvae fed both primary hosts or artificial diet.

Overall, 514 *D. texanus* unigenes were differentially expressed in larvae fed soybean compared to those fed the three diet treatments using fold change (FC) thresholds of ≥  ± 1.5 and False Discovery Rate (FDR) adjusted *p* values of ≤ 0.05. Soybean-fed larvae up-regulated 189 and 75 unigenes compared to larvae fed sunflower and giant ragweed, respectively (Fig. 1A); and down-regulated 127 and 111 unigenes compared to sunflower and giant ragweed, respectively (Fig. 1B). There were 46 and 19 unigenes that were commonly up and down-regulated in soybean-fed larvae compared to those fed both original plant hosts, respectively (Fig. 1A,B). Also, soybean-fed larvae differentially up-regulated and down-regulated 51 and 71 unigenes compared to those fed artificial diet, respectively (Fig. 1C,D).

**Figure 2:**
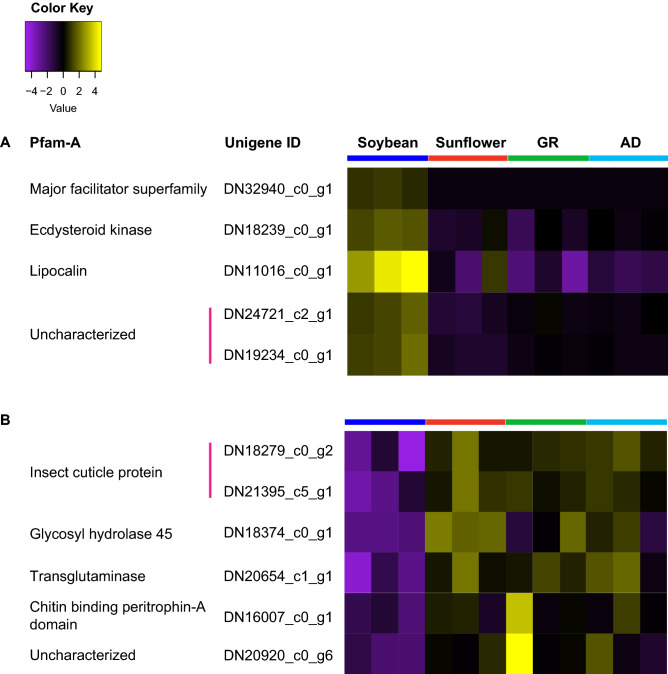
Heatmap of commonly (**A**) up-regulated and (**B**) down-regulated unigenes in *Dectes texanus* larvae fed soybean compared to those fed sunflower, giant ragweed (GR) and artificial diet (AD). Each row represents an individual unigene. Yellow and purple indicate high and low expression levels, respectively (Fold change >  ± 1.5, False Discovery Rate < 0.05). Pfam-A = Protein family-A domain; MFS = Major facilitator superfamily.

Of the unigenes differentially expressed by larvae fed soybean compared to those fed in sunflower and giant ragweed, only five and six unigenes were also up- and down-regulated compared to those fed artificial diet, respectively (Fig. 1C,D) while 41 and 13 unigenes were exclusively up-regulated and down-regulated, respectively, in soybean-fed larvae compared to those fed on sunflower and giant ragweed. Unigenes coding for proteins involved in transport of small hydrophobic molecules and solutes; and phosphorylation of ecdysteroids were among the five up-regulated unigenes in soybean-fed larvae compared to all diet treatments (Fig. 2A). Unigenes coding for insect cuticle proteins, GH45, chitin binding peritrophin-A domain, and transglutaminases were among the six down-regulated unigenes in larvae fed soybean compared to those fed both primary hosts and the artificial diet treatment (Fig. 2B).

Four molecular function GO categories were significantly (FDR < 0.05) enriched among the 41 commonly up-regulated unigenes in larvae fed soybean compared to those fed on sunflower and giant ragweed (Supplementary Table S5). These categories were oxidoreductase activity (GO:0016705), iron-ion binding (GO:0005506), tetrapyrrole binding (GO:0046906), and heme binding (GO:0020037), which included five unigenes coding for CYP enzymes belonging to the families CYP6 and CYP9. No GO categories were significantly enriched among the 13 commonly down-regulated unigenes in larvae fed soybean compared to those fed the other two plant hosts. GO categories significantly enriched in the differentially expressed unigenes in the soybean vs giant ragweed, soybean vs sunflower, and soybean vs artificial diet comparisons are listed in Supplementary Table S6.

**Figure 3:**
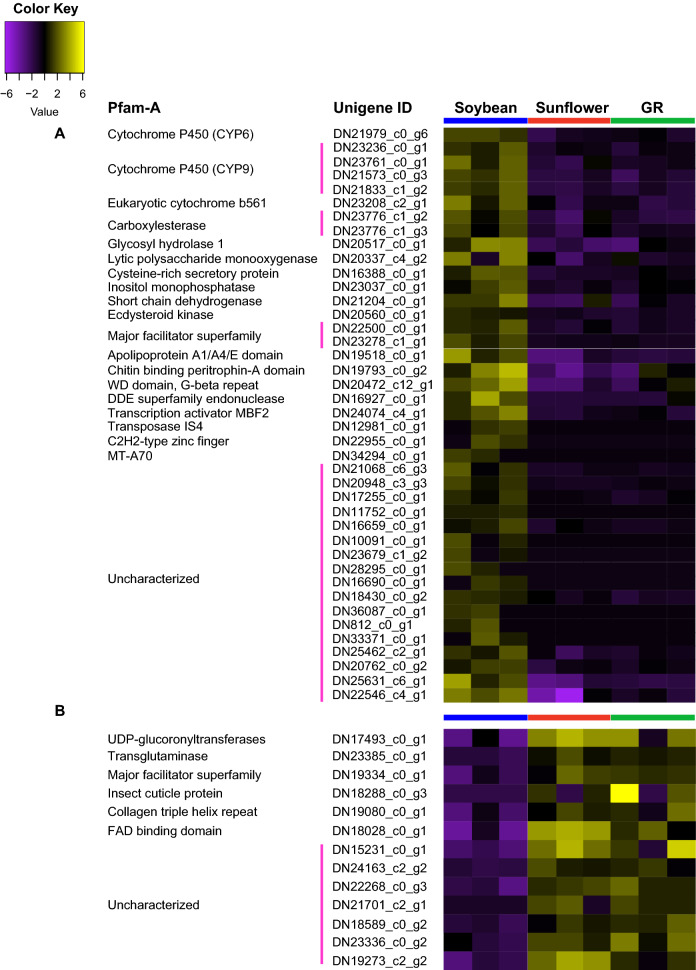
Heatmap of (**A**) up-regulated and (**B**) down-regulated unigenes in *Dectes texanus* larvae fed soybean compared to those fed sunflower and giant ragweed (GR). Each row represents an individual unigene. Yellow and purple indicate high and low expression levels, respectively (Fold change >  ± 1.5, False Discovery Rate < 0.05). Pfam-A = Protein family-A domain; CYP = Cytochrome P450; CarE = Carboxylesterase; MFS = Major facilitator superfamily; WD = Tryptophan-aspartic acid dipeptide; DDE = Aspartic acid-Aspartic acid-Glutamic acid motif; MBF2 = Multiprotein-bridging factor 2; IS4 = Insertion sequence 4 family; C2H2 = Cysteine- Cysteine -Histidine- Histidine motif; MT = Methyl-transferase; UGT = ; UDP-glucuronosyl-transferase; FAD = Flavin adenine dinucleotide.

Approximately 60% of the unigenes commonly up-regulated in larvae fed soybean compared to those fed sunflower and giant ragweed had significant BLASTp matches (e-values < 0.00001) to annotated proteins or contained known Pfam-A domains. Unigenes coding for CYPs, CarEs, and the major facilitator superfamily (MFS) proteins were the most represented protein families among the 41 up-regulated unigenes in larvae fed soybean (Fig. 3A). Protein-coding unigenes related to digestion and protein binding/transport included a GH1, a lytic polysaccharide monooxygenase, a short chain dehydrogenase, a chitin binding peritrophin-A, and an apolipoprotein (Fig. 3A). Unigenes coding for nuclear proteins were also represented among the up-regulated unigenes, and they included a transcription activator MBF2, a transposase IS4, a methyltransferase MT-A70, and a DDE endonuclease (Fig. 3A). The relationship of these unigenes in the use of soybean as a host is unclear, but higher expression of the MBF2 transcription factor may be associated to changes in gene expression in *D. texanus* larvae fed soybean. CYPs, CarEs, MFS transporters, and other unigenes related to digestion and detoxification may be associated with the utilization of soybean as a host by *D. texanus* third-instar larvae.

Among the 13 down-regulated unigenes in larvae fed soybean compared to the two primary hosts, 46% contained a significant match to a Pfam-A domain. These down-regulated unigenes contained the following protein families: an insect cuticle protein, a transglutaminase, a collagen triple helix repeat, an UGT, a MFS, and a FAD binding domain (Fig. 3B).

### K-means analyses of up-regulated and down-regulated unigenes in larvae fed soybean

In addition to the differential expression analysis, we also partitioned the data into clusters of unigenes that had similar expression profiles across the various diet treatments to identify genes that were expressed at higher or lower levels in soybean compared to the other diet treatments.

**Figure 4:**
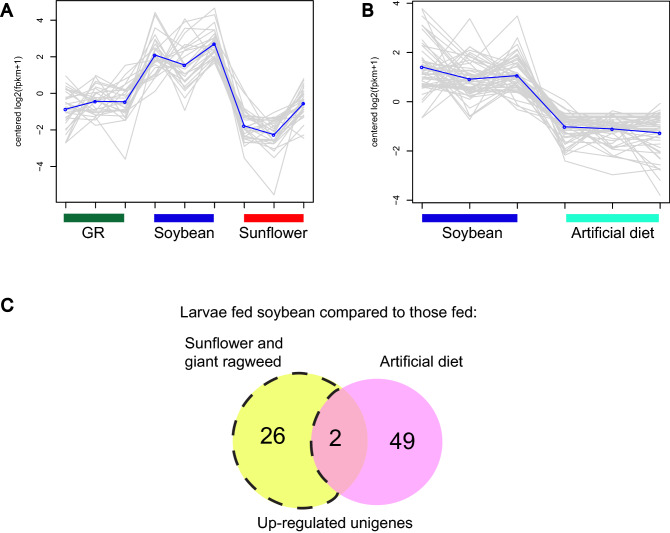
Expression profiles of up-regulated unigenes in *Dectes texanus* larvae fed different diet treatments (**A**) Unigenes with higher expression levels in larvae fed soybean compared to those fed sunflower and giant ragweed (**B**) Unigenes with higher expression levels in larvae fed soybean compared to those fed artificial diet and (**C**) Venn diagram of upregulated unigenes in larvae fed soybean compared to those fed sunflower, giant ragweed, and artificial diet. Clusters were constructed with differentially expressed genes showing similar expression patterns across all treatments (Fold change >  ± 1.5, False Discovery Rate < 0.05). GR = Giant ragweed.

**Figure 5:**
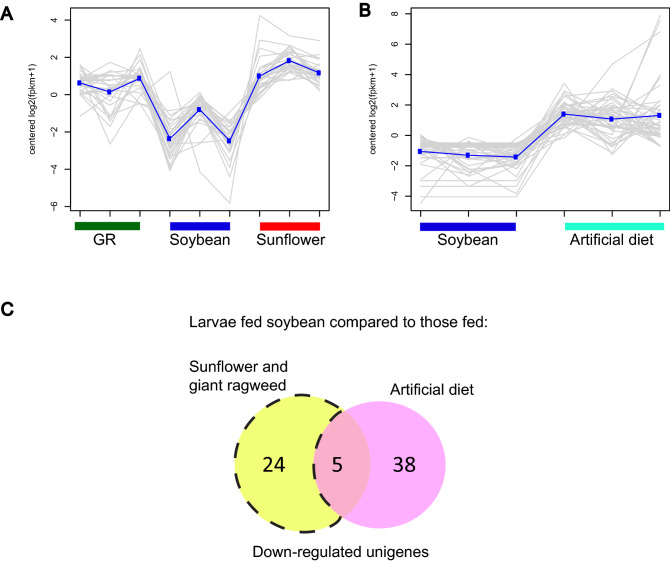
Expression profiles of down-regulated unigenes in *Dectes texanus* larvae fed different diet treatments (**A**) Unigenes with lower expression levels in larvae fed soybean compared to those fed sunflower and giant ragweed (**B**) Unigenes with lower expression levels in larvae fed soybean compared to those fed artificial diet and (**C**) Venn diagram of downregulated unigenes in larvae fed soybean compared to those fed sunflower, giant ragweed, and artificial diet. Clusters were constructed with differentially expressed genes showing similar expression patterns across all treatments (Fold change >  ± 1.5, False Discovery Rate < 0.05) GR = Giant ragweed.

Using this method, 28 and 51 unigenes were highly expressed in soybean-fed larvae compared to larvae fed sunflower and giant ragweed or artificial diet, respectively (Fig. 4A,B). Further, 29 and 43 unigenes were expressed at lower levels relative to those fed on the two primary hosts or on artificial diet, respectively (Fig. 5A,B). Only two and five unigenes were consistently up- or down-regulated in soybean fed larvae compared to all three diet treatments, respectively (Figs. 4C, 5C); whereas only 26 and 24 unigenes were expressed at higher or lower levels exclusively in soybean fed larvae compared to those fed on sunflower and giant ragweed (Figs. 4C, 5C). This analysis allowed us to detect additional unigenes that were expressed at higher/lower levels in soybean that were not detected in expression analyses presented in the previous section, as well as unigenes with expression profiles of interest that were detected in both analysis methods.

Two unigenes, one lacking Pfam-A domain and BLASTp matches and another with a FAD-binding domain, were exclusively identified in the K-mean analysis as differentially expressed in soybean fed-larvae compared to those fed sunflower, giant ragweed, and artificial diet exclusively (Fig. 7A,B). Additionally, induced unigenes coding for a CYP6, three GH28, two MFS, and an AMP-binding enzyme in soybean-fed larvae compared to those fed both primary plant hosts were found in the K-means analysis, only (Fig. 7A). Other down-regulated unigenes in larvae fed soybean compared to those fed sunflower and giant ragweed and found exclusively in the K-means analysis included a CarE, a GH28, a trypsin, two MFS, a hemocyanin, chitin binding perithrophin-A domain and a TGF- beta propeptide (Fig. 7B).

Five molecular function GO categories were significantly enriched among the 26 unigenes that were more highly expressed in soybean-fed larvae compared to those fed on the two primary hosts (Supplementary Table S7). The polygalacturonase activity GO term (GO:0004650) was among the enriched categories and was not detected in the differential expressional expression analysis with edgeR. This GO term included three GH28 unigenes. As in the differential expression analysis, no GO categories were significantly enriched among the unigenes that were expressed at lower levels in soybean fed larvae compared to those fed on sunflower and giant ragweed.

**Figure 6:**
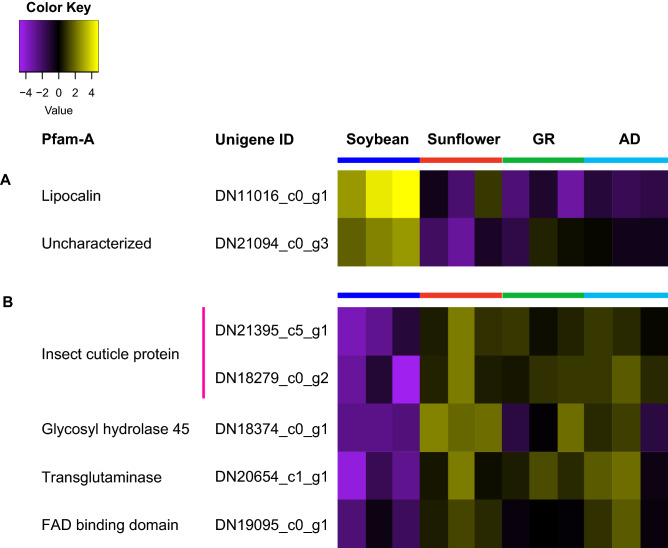
Heatmaps of unigenes expressed at (**A**) higher and (**B**) lower levels in larvae fed soybean compared to those fed sunflower, giant ragweed, and artificial diet by K-means analyses. Each row represents a separate unigene. Yellow and purple indicate high and low expression levels, respectively (Fold change >  ± 1.5, False Discovery Rate < 0.05). Pfam-A = Protein family-A domain; FAD = Flavin adenine dinucleotide.

An unigene containing a lipocalin protein domain was the only commonly up-regulated unigene in soybean-fed larvae compared to those fed the three other diet treatments in both analysis methods (Fig. 6A). This unigene had a BLASTp match to a fatty acid-binding protein (FABP) from *A. glabripennis*. Lipocalins and FABPs are involved in transport of small hydrophobic molecules across extra- and intra-cellular membranes^79^. Four commonly down-regulated unigenes in soybean-fed larvae compared to those fed sunflower, giant ragweed, and artificial diet in both analysis methods included two unigenes coding for insect cuticle proteins, one coding for a GH45, and one coding for a transglutaminase (Fig. 6B).

**Figure 7:**
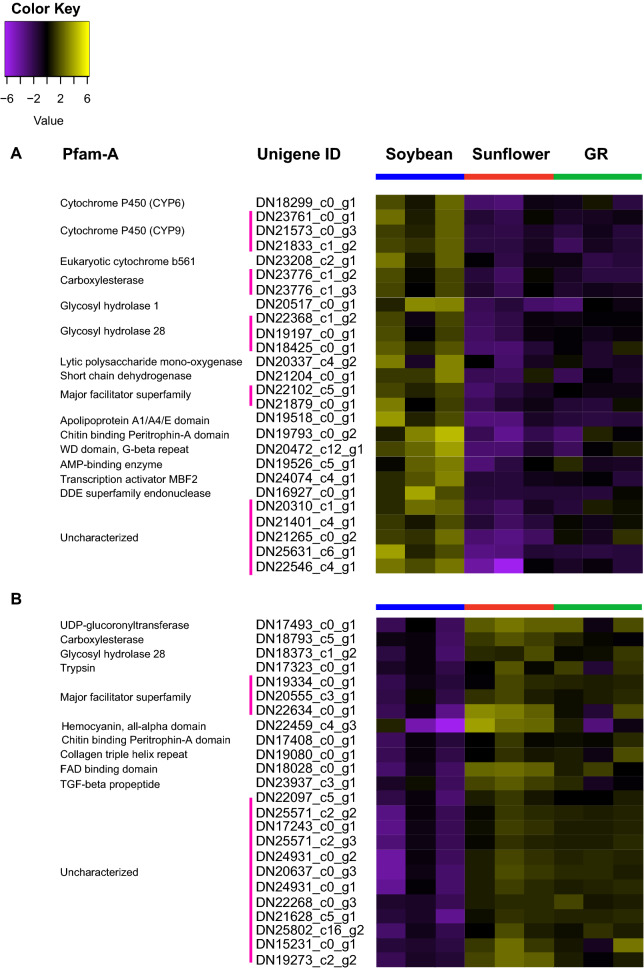
Heatmaps of unigenes expressed at (**A**) higher and (**B**) lower levels in larvae fed soybean compared to sunflower and giant ragweed by K-means analyses. Each row represents a separate unigene. Yellow and purple indicate high and low expression levels, respectively (Fold change >  ± 1.5, False Discovery Rate < 0.05). Pfam-A = Protein family-A domain; CYP = Cytochrome P450; CarE = Carboxylesterase; MFS = Major facilitator superfamily; WD = Tryptophan-aspartic acid dipeptide; AMP = Adenosine monophosphate; MBF2 = Multiprotein-bridging factor 2; DDE = Aspartic acid-Aspartic acid-Glutamic acid motif; UGT = UDP-glucuronosyl-transferase; FAD = Flavin adenine dinucleotide; TGF = Transforming growth factor.

Protein families involved in biotransformation of plant allelochemicals, digestion of plant cell walls, transport of small lipids, and protein binding represented 42.3% of the 26 unigenes highly expressed in soybean-fed larvae compared to those fed in the two primary hosts in both analysis methods (Fig. 4, 7A). Unigenes coding for a WD domain-G-beta repeat, a transcription activator MBF2, a DNA-binding endonuclease, an UGT, a MFS, a collagen triple helix repeat, and a FAD binding domain were among the up- and down-regulated unigenes in larvae fed soybean compared to sunflower and giant ragweed in the EdgeR and K-means analyses (Fig. 7A,B).

## Discussion

Albeit soybean is a new and challenging host for *D. texanus*, gene expression changes of third-instar larvae feeding in soybean compared to sunflower and giant ragweed were relatively limited in number and largely involved unigenes coding for enzymes associated with detoxification and digestion. Differences between host plant defense compounds produced by the different hosts in this study may be linked to differential expression of genes involved in biotransformation of host plant defenses. Although it is unknown the types and concentration of plant defense chemicals in the stems of the host plants used in this study, sunflower coumarins (scopoletin, ayapin and scopoline) have antifungal properties and deter *Zygogramma exclamationis* Fabricius from feeding, and soybean isoflavones (daidzin, genistin and afrormosin) and pterocarpans (glyceollins) confer resistance to insects^80–85^. The effects of giant ragweed defense compounds against insects are unknown, but their monoterpenes and sesquiterpenes found in essential oils have antimicrobial properties that inhibit germination of wheat, lettuce, watermelon and tomato^86–88^. Unigenes coding for CYPs belonging to the CYP6 and CYP9 families and two CarEs were induced in soybean fed larvae compared to those fed both primary hosts. Known members of the CYP6 family are linked to detoxification of plant allelochemicals in other insect species and are induced by and metabolize flavones, coumarins, and sesquiterpenes in *Helicoverpa zea* Boddie*, Spodoptera litura* Fabricius*,* and *S. frugiperda*^89–92^*.* Additionally, members of the CYP9 families are involved in insecticide and plant allelochemical detoxification in other insect species^93–97^. For example, CYP9A40 is induced by quercetin, cinnamic acid, deltamethrin and methoxyfenozide in *S. litura*, and it is involved in the detoxification of these compounds^98^. Although it is unknown which substrates the enzymes of the CYP9 and CYP6 families in *D. texanus* are capable of acting on, they may be relevant for use of soybean as a host due their ability to detoxify plant defensive compounds in other insect species The two up-regulated unigenes coding for CarEs had a significant BLASTp match to proteins annotated as an *esterase 1* and an *esterase S* from *Schizaphis graminum* Rondani and *Drosophila virilis* Sturtevant, respectively, and they belonged to functional clade A. CarEs belonging to clade A have been previously associated with organophosphate, carbamate, malathion, fipronil, and cyhalothrin detoxification in insects^64,99–101^. Although, little is known about the dietary functions of CarEs in clade A, it has been suggested that this clades may be relevant for the adaptation of coleopterans to their ecological niches^63,64^. These CarEs may be involved in the hydrolysis of ester bonds in pectin and hemicellulose^102^, and detoxification of plant secondary metabolites in *D. texanus* larvae.

In the comparisons between soybean-fed larvae and those fed both primary plant hosts or artificial diet, an unigene coding for an UGT411 in *D. texanus-*fed soybean was down-regulated whereas unigenes coding for CYPs and CarEs were up-regulated in this treatment. It has been suggested that UGT411 expansion is associated with wide host range and adaptation in *A. glabripennis*^43^*.* Thus, down-regulation of these genes may be attributed to differences in composition of defensive compounds in soybean relative to the primary plant hosts.

Unigenes coding for a GH1 and a lytic polysaccharide monooxygenase were also up-regulated in soybean-fed larvae compared to those fed both primary hosts. The combined action of lytic monooxygenases and GH1 hydrolases may be important for boosting digestion of plant cell walls by hydrolysis and oxidation of glycosidic bonds, and their expression may be up-regulated in soybean-fed larvae in response to high carbohydrate content in soybean stem pith^42^. While glycoside hydrolases responsible for digesting plant cell wall polysaccharides have been identified in a taxonomically diverse range of beetle species, polysaccharide monooxygenases coded by insects have been comparatively less well studied; however, *in vitro* assays in *Thermobia domestica* Packard showed that these enzymes are potentially important for digesting cellulose^103^*.* Although the unigene coding for a GH1 has a significant BLASTp match to a *myrosinase 1*, it is most likely acting as β-glucosidase to degrade di- and tri-saccharide sugars found in plant tissues or act on other plant secondary compounds^104,105^, since glucosinolates are not known to occur in soybeans or in plant hosts from the family Asteraceae. Three up-regulated unigenes coding for polygalacturonases (GH28) were also identified in soybean-fed larvae in the K-means and GO enrichment analyses. These genes are probably involved in the hydrolysis of glycosidic bonds of de-esterified pectins^57^. An up-regulated unigene coding for a chitin binding peritrophin-A was also identified in soybean-fed larvae using both methods of gene expression analyses. This unigene most likely codes for a GH18 (chitinase) based on significant BLASTp matches to probable *Drosophila melanogaster* Meigen and *A. glabripennis* chitinases. This domain is found in peritrophic matrix proteins and chitinases. This unigene may be involved in maintenance of the *D. texanus* larval peritrophic matrix integrity or digestion of conspecifics as *D. texanus* larvae are known to be cannibalistic^106^.

Differential expression of unigenes coding for MFS transporters were detected in soybean compared to those fed sunflower and giant ragweed. Upregulated unigenes containing the MFS protein domain were closely related to beetle proteins coding for sugar porter and vesicular neurotransmitter transporter families with bootstrap values higher than 95% (Supplementary Fig. S12 and S13). The three up-regulated and one down-regulated *D. texanus* unigenes belonging to the sugar porter family had significant BLASTp matches to proteins annotated as facilitated trehalose transporters in *T. castaneum*. Higher expression of MFS transporters and sugar porters has been reported in insects after feeding on different host and non-host plants^10,17,18,20,107,108^. Although these transporters can have a variety of different functions, it has been suggested that sugar porters mediate sugar uptake from phloem in *Nilaparvata lugens* Stål^109^ and maintain osmotic balance in *Acyrthosiphon pisum* Harris^110^. *Dectes texanus* sugar porters may mediate concentration of trehalose in their bodies since trehalose is the major carbohydrate found in *D. texanus* larvae collected from sunflower^111^. Additionally, the high carbohydrate content in soybean stem pith^42^ may stimulate induction of sugar porters in *D. texanus* larvae fed soybean. Interestingly, total carbohydrate concentration was not significantly different between larvae collected from sunflower and soybean in a more recent study^42^. The up-regulated *D. texanus* unigene belonging to the vesicular neurotransmitter transporter family had a significant BLASTp match to *T. castaneum* synaptic vesicle glycoprotein 2B. Although, the function of this protein is unknown in insects, transcripts of the synaptic vesicle glycoprotein 2B were highly abundant in the Malpighian tubules of *Zophobas morio* Fabricius larvae compared to the fat body tissue^112^. Synaptic vesicle glycoproteins 2 (A-C) are proposed to transport sugars and neurotoxins rather than neurotransmitters across the synaptic vesicle membrane and stabilize neurotransmission by supporting the vesicular structure and mediating neuronal calcium dynamics^113^. It remains to be investigated which compound(s) from soybean promote up-regulation of this vesicular neurotransmitter transporter in *D. texanus*.

Only a single unigene coding for a FABP was exclusively up-regulated in soybean compared to the other three diet treatments . Lipocalins are proteins involved in the transport of small hydrophobic molecules across membranes and are linked to diverse physiological processes including olfaction, coloration, prostaglandin synthesis, immune response, and cell homoeostasis^114^. Although the function of FABPs is less well defined, they are proposed to be involved in intracellular transport of fatty acids from the cell membrane to the mitochondria and in regulating free fatty acid concentration in the cytosol^79^. Differential expression of genes containing lipocalin protein domains was previously reported in *T*. *urticae* feeding on maize, cotton, soybean, lima bean and tomato for at least five generations compared to those fed common bean^107^. Induction of lipocalins in *T. urticae* may be associated with sequestration of hydrophobic allelochemicals and protection from the oxidative stress response of its host plants^107,108^. Although, it is unknown which metabolic process is affected by up-regulation of this FABP in *D. texanus* larvae fed soybean, they could be associated with the maximization of lipid transport and storage when feeding on a host (soybean stem pith) with low lipid content^42^. Lipocalins may be important for the development of *D. texanus* third-instar larvae feeding on soybean.

Overall, *D. texanus* third-instar larvae exhibited a narrow transcriptomic response to feeding on soybean compared to those fed sunflower, giant ragweed and artificial diet. Only 3.5% of *D. texanus* unigenes were differentially expressed between soybean-fed larvae and the diet treatments, and no major impacts on the expression levels of genes coding for enzymes involved in core metabolic pathways were detected when feeding on soybean. Up-regulation of unigenes involved in digestion, biotransformation of plant allelochemicals and transport of molecules most likely is important for *D. texanus* in using soybean as host. FABP (lipocalins) and MFS transporters, in conjunction with apolipoproteins, may be involved in regulating levels and transport of small carbohydrates and lipids in *D. texanus* larvae fed soybean^108^. Up-regulation of these unigenes may be associated with high carbohydrate and low lipid content in soybean stem piths^42^. Functional validations of the unigenes coding for CYPs, CarEs, GHs, lytic monooxygenases, sugar porters, lipocalins, and synaptic vesicle glycoproteins are required to understand their contribution in the process of adaptation of this species to soybean. Gene editing and silencing technologies can be used to validate these genes and considered for management of *D. texanus* as current commercial insecticides fail to control the larval stage feeding inside the stems^36,115–117^*.* Also, this study provides valuable information for the development of soybean cultivars resistant to *D. texanus* as it suggests possible unigenes that may be involved in *D. texanus* adaptation to soybean.

## Methods

### Estimation of *D. texanus* genome size

The estimated genome size for *D. texanus* was determined with flow cytometry from seven females and eight males collected from a laboratory colony reared on pink bollworm artificial diet at Kansas State University (KSU), Manhattan, KS, USA. Single *D. texanus* male or female individuals were prepared for genome size estimation^118^. In brief, individual heads were separately placed into a 2 ml Kontes Dounce tissue grinder vials containing 1 ml of Galbraith buffer^119^. An internal standard (1C = 328 Mbp) consisting of the head of a female *D. virilis* was included in the analysis. Samples were then ground with 15 strokes of the “B” pestle, filtered through a 40 u nylon filter, stained with 25 µg/ml propidium iodide (PI), and stored at − 20 °C for at least 30 min. After storage, each sample and standard were scored for the relative PI fluorescence of diploid nuclei using a Partec CyFlow flow cytometry equipped with a Cobalt Samba laser emitting at 532 nm. At least 2000 nuclei were scored for each peak, with the coefficient of variation for each sample less than 3.0. Genome size (1C) was estimated as the genome size standard*mean PI–fluor beetle/ mean PI–fluor standard. An average genome size was estimated using the seven *D. texanus* females and eight males, respectively, and an overall average was estimated using both sexes. The estimation of *D. texanus* genome size was performed at the Department of Entomology, Texas A&M AgriLife Research, College Station, TX USA.

### Plant materials

Seeds of the *D. texanus-*susceptible soybean genotype K07_1544, common sunflower and giant ragweed were provided by the KSU Soybean Breeding Program and Weed Ecology Lab, respectively. Sunflower and giant ragweed seeds were pre-germinated in soil-filled flats in a cold room (4 °C) for 21 d and moved to the greenhouse for germination 7 d before planting in the field. Giant ragweed seeds did not germinate. Thus, seedlings were collected from giant ragweed populations around the experimental plots at the KSU Ashland Bottoms Research Station, near Manhattan, KS, and transplanted to cylindrical pots (10 cm wide × 8.5 cm deep) with soil 7 d before planting in the field. Giant ragweed seedlings were identified based on the spoon-shaped cotyledons and first true leaf in the greenhouse before planting in the field.

### Experimental design for RNAseq analyses

The treatments were arranged in nine 3 × 3 m plots at the KSU Ashland Bottoms Research Station where each plot consisted of four 2.3 m long rows with five plants per row spaced 30 cm apart, for a total of 20 plants per plot. All plants in a plot were of the same species to prevent any host bias during oviposition by *D. texanus*. Three replicate plots were planted for each plant species. Soybean seeds, and sunflower and giant ragweed seedlings were hand-planted about 2.5 cm and 10 cm deep, respectively, in late May 2017.

Plants within each plot were caged 21 d after planting in 3 × 3 × 1.8 m polyvinyl chloride (PVC) frame covered with mosquito mesh to prevent other insects from colonizing the plants and prevent beetles from escaping. Caged plants were infested with unsexed *D. texanus*-adults collected from nearby soybean fields at the research station at a rate of four beetles per plant 7 d after they were caged. Adult *D. texanus* are sexually monomorphic, so a sex ratio of 1 female to 1 male was assumed and adults were evenly distributed in each cage/plot. Adults were allowed to mate and lay eggs for a period of 21 d. After this time period, *D. texanus* larvae were collected by cutting three plants per cage at soil level and splitting the stems. There were no indications of female oviposition biases related to host of provenance. Previous data showed that *D. texanus* adults infest and lay eggs on soybean or sunflower regardless of their species-host of origin^39,40^. Three third-instar larvae collected from different plants within the same plot were pooled together and represent one biological replicate. A total of three biological replicates per plant species were collected for analysis. Larval instar^37^ was estimated based on larval head capsule width, and samples were stored at − 80 °C until RNA extraction.

*Dectes texanus* third instar-larvae fed pink bollworm artificial diet (Frontier Scientific Services, Newark, DE USA) since egg hatch were also collected for RNA extraction. Previous studies indicated that *D. texanus* can be successfully reared to maturity on this artificial diet where the main ingredients are wheat germ, cellulose and casein^29^. *Dectes texanus* field-collected adults were provided green beans as an oviposition substrate and kept inside mite-proof cages (20.2 wide × 20.8 long × 20.2 tall cm, 35-micron mesh). These adults were collected from the same soybean fields as the adults used in the field experiment described above. Pods were dissected to harvest eggs that were then stored on petri dishes with moist filter paper until eclosion (4 d later). After egg hatch, larvae were fed artificial diet as indicated in Hatchett’s rearing protocol^29^. *Dectes texanus* adults, eggs and larvae were kept in mite-proof cages inside a Thermo Scientific growth chamber (27 °C, 14L:10D) at the KSU Department of Entomology, Manhattan, KS. Three biological replicates were collected for analysis, with each replicate consisting of three pooled third-instar larvae collected from within the same cage. Larval head capsules were measured to estimate larval instar^37^ before storage inside a -80 °C freezer until RNA extraction.

### RNA extraction and mRNA sequencing

Total RNA was extracted from whole bodies of *D. texanus* by using RNeasy Plus Mini Kit (Qiagen, Hilden, Germany) according to the manufacturer’s protocol with the addition of a DNA elimination step. Three biological replicates were prepared for each of the four diet treatments (three plant species and one artificial diet). RNA quality and quantity were measured with an RNA 6000 Nano Assay on an Agilent 2100 Bioanalyzer (Agilent, Santa Clara, CA USA) and a NanoDrop ND-ONE Spectrophotometer (Thermo Fisher Scientific, Waltham, MA USA), respectively, before construction of cDNA libraries. RNA was of high quality and showed no degradation based on electropherograms and spectrophotometry. Separate cDNA libraries for each biological replicate per diet treatment were constructed using 2 ug of total RNA with a TruSeq Stranded mRNA Library Preparation Kit v2 (Illumina, San Diego, CA USA) according to the manufacturer’s instructions that included indexed-adaptor ligation and oligo-dT beads to capture poly(A) tails. Libraries were amplified by PCR for 8 cycles with a KAPA-Library Quantification Kit for Illumina (KAPA Biosystems, Wilmington, MA USA) using a PE9700 thermal cycler (Perkin Elmer, Waltham, MA USA). cDNA library quality and quantity were measured with the DNA High Sensitivity Assay on an Agilent 2100 Bioanalyzer (Agilent, Santa Clara, CA USA) and qPCR on an Applied Biosystems Step One instrument (Thermo Fisher Scientific, Waltham, MA USA), respectively. The average cDNA lengths ranged from 482 to 515 bp for the 12 libraries, and the overall average was 497 bp. Indexed Illumina libraries were combined into a single library pool and sequenced as 2 × 100 PE reads in one lane of a 2-lane Rapid Flowcell on an Illumina HiSeq2500. Illumina library preparation and sequencing were conducted at Purdue University Genomics Core Facility, West Lafayette, IN USA.

### De novo transcriptome assembly

Reads were trimmed to remove low quality bases (PHRED score < 20) and residual Illumina adapters using the program Trimmomatic^120^ v.0.38, and reads shorter than 30 nt after quality trimming were discarded. FastQC (https://www.bioinformatics.babraham.ac.uk/projects/fastqc/) was used to assess the quality of the reads before and after quality filtering.

A de novo transcriptome assembly was performed using the trimmed and filtered reads from all twelve *D. texanus* samples with Trinity v.2.6.5^121^ including default settings and in silico normalization. Afterwards, reads from each sample were mapped back to the raw transcriptome assembly using the align_and_estimate_abundance.pl^122^ script with bowtie2^123^ for read mapping and RSEM^124^ for abundance estimation. Transcripts with < 0.1 transcripts per million mapped reads (TPM) or transcripts representing < 5% of the expression value of the dominant isoform for each unigene were removed from the transcriptome assembly because they lacked sufficient read depth to support their inclusion in the final assembly. Putative open reading frames (ORFs) of at least 100 amino acid residues in length were identified using Transdecoder v.5.0.2 (https://github.com/TransDecoder/TransDecoder/releases). The identification of ORFs was facilitated using BLASTp (ncbi-blast v.2.6.0 +) searches against the sprot database (downloaded on February 5, 2018) and hmmer searches against the Pfam-A database. The single highest scoring ORF with a BLASTp match to the sprot database or a Pfam domain for each transcript was retained using the single_best_orf, retain_pfam_hits and retain_blastp_hits options in Transdecoder. Finally, transcripts containing no open reading frames were removed from the assembly and only those that likely coded for proteins were included in our downstream annotation and differential expression analyses.

Predicted ORFs were searched against the non-redundant protein database (downloaded on October 4, 2018) using BLASTp to identify any potential plant or microbial transcripts in the assembly. In brief, the top five BLASTp matches with e-values ≤ 0.00001 were retained for each predicted coding region and taxonomic classifications were carried out using MEGAN's^125^ least common ancestor algorithm. Transcripts derived from plants, viruses, or microbial taxa were considered contaminants and were removed from the assembly prior to annotation and differential expression analyses. Remaining transcripts were functionally annotated using Trinotate^126^. Predicted proteins were annotated using BLASTp searches against the sprot database (downloaded on March 1, 2018); Pfam-A domains were predicted using HMMER^127^ and the Pfam-A database (downloaded on March 1, 2018); signal peptides were predicted using signalP^128,129^, and transmembrane regions were predicted using tmHMM^130^.

Additionally, *D. texanus* unigenes were assigned to KEGG orthology terms using KAAS (KEGG automatic annotation server)^131^ with the bi-directional best hit method for partial genomes and the *T. castaneum*^132^ and *D. ponderosae*^133^ genomes as references. KEGG orthology assignments were compared to KEGG annotations from the *A. glabripennis* genome using the predicted proteome from the assembly version GCA_000390285.2 downloaded from NCBI. Each KO term was counted once in the *D. texanus* transcriptome and in the other insect genomes (Supplementary Table S4). Assessment of *D. texanus* unigenes-transcriptome completeness was performed using the program BUSCO (Benchmarking Universal Single-Copy Orthologs) v3 against the Insecta ODB9 gene set^134,135^.

### Phylogenetic analyses

Phylogenetic analyses were conducted to assign *D. texanus* unigenes coding for putative CarEs, UGTs, GSTs, CYPs, ABC transporters, and lipocalin to families and subfamilies using *T. castaneum*^63,64,67,77,78,136^ and *A. glabripennis*^43^ protein sequences retrieved from NCBI as references. Only proteins that encoded complete ORFs were included in the analysis and microsomal GSTs were excluded from the phylogenetic analysis. In brief, *D. texanus, A. glabripennis*, and *T. castaneum* amino acid sequences were aligned with MUSCLE (default parameters) using MEGAX^137^. The alignments were used to construct maximum-likelihood (ML) trees using RAxML-HPC2 Workflow on XSEDE (version 8.2.12)^138^ in CIPRES Science Gateway^139^ with the following parameters: Jones–Taylor–Thornton (JTT) protein substitution matrix, GAMMA model of rate heterogeneity, 1000 bootstrap iterations, default seed value (12,345) for multi-parametric bootstrapping, branch lengths for mean substitutions per site estimation, and Majority Rule consensus tree computation with a ≥ 50% bootstrap threshold. Majority Rule consensus trees were viewed and edited on iTOL (Version 5.6.3)^140^.

MFS and ABC sequences from *D. texanus* and *A. glabripennis* were classified into families by BLAST searches against the Transporter Classification Database (TCDB)^141^ and distant homology evaluations using the Global Sequence Alignment Tool (GSAT)^142^ set to 500 shuffles and with a Standard Z-score (SD) ≥ 9 threshold (Supplementary Table S8 and S9). A SD value ≥ 9 corresponds to a probability of 10^–19^ of achieving sequence similarity by chance and is considered sufficient to establish homology among putative full-length proteins^143,144^. Sequence similarity searches and GSAT alignments were performed on the TCDB webpage (http://www.tcdb.org/analyze.php)141,142. MFS-phylogenetic trees were constructed as mentioned above.

### Differential expression analyses

After removing non-coding, low abundance and contaminant transcripts from the assembly, reads from the 12 libraries were re-aligned to the filtered transcriptome assembly, individually, using bowtie2 and RSEM as described above. RSEM counts were concatenated into two count matrixes. The first matrix contained all samples from larvae collected from the three plant hosts, and the second contained samples from soybean and artificial diet-fed larvae only. Differential expression analyses were conducted at the unigene level using edgeR^145^ and unigenes differentially expressed among the three plant treatments were identified using the first count matrix. Unigenes differentially expressed between soybean and artificial diet were also identified using the second matrix. Partitioning the samples in two matrixes helped to identify unigenes that were exclusively differentially expressed in the soybean treatment and were likely influenced by defensive compounds associated with this host.

Read counts were normalized using trimmed means (TMM) and variances were estimated using tagwise dispersions. Only transcripts with counts per million (CPM) values greater than one in at least two samples were tested for differential expression. Pairwise comparisons were used to identify unigenes that were differentially expressed in at least one sample using Fisher's Exact test. Unigenes with FC ≥ 1.5 or ≤ -1.5 (relative log FC ≥ 0.6) and FDR corrected *p* values ≤ 0.05 were considered differentially expressed. Gene ontology (GO) enrichments were performed using GOseq^146^, and the entire list of unigenes with CPM > 1 in at least two samples were used as a reference to determine enrichment. Nodes containing less than five unigenes were excluded from the GOseq analysis to control false discovery rate. K-means analysis^122^ was performed to identify groups of *D. texanus* unigenes with similar expression patterns across the three plant diet treatments. Unigenes that were upregulated or downregulated in soybean in comparison to the two primary plant hosts and the artificial diet were also identified to determine which unigenes were specifically differentially expressed when fed soybeans.

The computing for this project was performed on the Beocat Research Cluster at KSU, Manhattan, KS USA. Raw paired end reads for each sample were deposited on NCBI BioProject PRJNA698984. SRA accession numbers are listed in Supplementary Table S1.

### Ethical statement

All international, national and institutional guidelines applicable to use and feed crop plants to insects were carefully followed in this study.

## Additional information

This manuscript is contribution number 21–203-J of the Kansas Agricultural Experiment Station. The use of trade names is for the purposes of providing scientific information only and does not constitute endorsement by the United States Department of Agriculture. The USDA is an equal opportunity employer.

## Supplementary Information

Supplementary Information 1.

Supplementary Information 2.

Supplementary Information 3.

Supplementary Information 4.
