# Supplementary material for: Comparative analyses of transcriptional responses of Dectes texanus LeConte (Coleoptera: Cerambycidae) larvae fed on three different host plants and artificial diet
Source: Sci Rep. 2021 Jun 1;11:11448. doi: 10.1038/s41598-021-90932-x (PMC8169664; doi:10.1038/s41598-021-90932-x)
Supplement: Supplementary file 2 — Supplementary Information 2. [file 41598_2021_90932_MOESM2_ESM.docx]

**Supplementary Figure S1. Taxonomic classification of highest scoring BLASTp matches. 93.6% of *Dectes texanus* protein coding transcripts match orders in the class Insecta.**

**Supplementary Figure S2. KEGG pathway classification of *Dectes texanus* transcriptome and *Anoplophora glabripennis* genome**. (A) Organismal systems, (B) Cellular processes, (C) Environmental information processing, (D) Genetic information processing, (E) Metabolism.

**Supplementary Figure S3. Number of *Dectes texanus* protein families and *Anoplophora glabripennis* genes coding for (A) glycoside hydrolases (GH) and (B) detoxification enzymes.**

**Supplementary Figure S4. Percentage of unigenes coding for glycoside hydrolase (GH) families in the *Dectes texanus* transcriptome.**

**Supplementary Figure S5. Phylogenetic relationships among the *Dectes texanus, Anoplophora glabripennis* and *Tribolium castaneum* cytochrome P450s (CYPs).** A maximum likelihood analysis of *D. texanus* CYP unigenes with a complete ORF was performed using RAxML-HPC2 Workflow on XSEDE in CIPRES Science Gateway with a Jones-Taylor-Thornton (JTT) protein substitution matrix and 1000 bootstrap replicates. The resulting Majority Rule consensus tree with a >= 50% bootstrap threshold was drawn using iTOL. Bootstrap values are represented in purple circles. The four clans CYP2, CYP3, CYP4 and mito are shown in different colors. Scale bar indicates branch lengths. *Leptinotarsa decemlineata* CYPs were included to resolve branches. *Dectes texanus* sequences are presented in bold.

**Supplementary Figure S6. Phylogenetic relationships among the *Dectes texanus, Anoplophora glabripennis* and *Tribolium castaneum* carboxylesterases (CarEs).** A maximum likelihood analysis of *D. texanus* CarE unigenes with a complete ORF was performed using RAxML-HPC2 Workflow on XSEDE in CIPRES Science Gateway with a Jones-Taylor-Thornton (JTT) protein substitution matrix and 1000 bootstrap replicates. The resulting Majority Rule consensus tree with a ≥ 50% bootstrap threshold was drawn using iTOL. Bootstrap values are represented in purple circles. The three CarE functional clades are highlighted in different colors. *Leptinotarsa decemlineata* CarEs were included to resolve branches. Scale bar indicates branch lengths. *Dectes texanus* sequences are presented in bold.

**Supplementary Figure S7. Phylogenetic relationships among the *Dectes texanus, Anoplophora glabripennis* and *Tribolium castaneum* UDP-glucuronyl transferases (UGTs).** A maximum likelihood analysis of *D. texanus* UGT unigenes with a complete ORF was performed using RAxML-HPC2 Workflow on XSEDE in CIPRES Science Gateway with a Jones-Taylor-Thornton (JTT) protein substitution matrix and 1000 bootstrap replicates. Bootstrap values are represented in purple circles. The resulting Majority Rule consensus tree with a ≥ 50% bootstrap threshold was drawn using iTOL. Scale bar indicates branch lengths. *Leptinotarsa decemlineata* UGTs were included to resolve branches. *Dectes texanus* sequences are presented in bold. UGT411 and 412 were formerly known as families UGT352 and 353 in *A. glabripennis* and other Cerambycids (New names approved by the UGT nomenclature committee on February 2, 2021).

**Supplementary Figure S8. Phylogenetic relationships among the *Dectes texanus, Anoplophora glabripennis* and *Tribolium castaneum* glutathione *S*-transferases (GSTs).** A maximum likelihood analysis of *D. texanus* GST unigenes with a complete ORF was performed using RAxML-HPC2 Workflow on XSEDE in CIPRES Science Gateway with a Jones-Taylor-Thornton (JTT) protein substitution matrix and 1000 bootstrap replicates. The resulting Majority Rule consensus tree with a ≥ 50% bootstrap threshold was drawn using iTOL. Bootstrap values are represented in purple circles. Six cytosolic, one microsomal and one unknown (UN) classes are highlighted in different colors. *Dectes texanus* and *A. glabripennis* microsomal GSTs were excluded from the analysis. Scale bar indicates branch lengths. *Dectes texanus* sequences are presented in bold.

**Supplementary Figure S9. Phylogenetic relationships among the *Dectes texanus, Anoplophora glabripennis* and *Tribolium castaneum* ATP-binding cassette transporters (ABCs).** A maximum likelihood analysis of *D. texanus* ABC unigenes with a complete ORF was performed using RAxML-HPC2 Workflow on XSEDE in CIPRES Science Gateway with a Jones-Taylor-Thornton (JTT) protein substitution matrix and 1000 bootstrap replicates. The resulting Majority Rule consensus tree with a ≥ 50% bootstrap threshold was drawn using iTOL. Bootstrap values are represented in purple circles. ABC clusters are highlighted in different colors. Scale bar indicates branch lengths. *Dectes texanus* sequences are presented in bold.

**Supplementary Figure S10. Heatmap of *Dectes texanus* differentially expressed unigenes (fold change > ± 1.5, False Discovery Rate < 0.05) in larvae fed soybean (blue), sunflower (red), or giant ragweed (green).** Each row represents a separate unigenes. Yellow and purple indicate high and low expression levels, respectively.

**Supplementary Figure S11. Heatmap of *Dectes texanus* differentially expressed unigenes (fold change > ± 1.5, False Discovery Rate < 0.05) in larvae fed soybean (red) or artificial diet (AD, blue).** Each row represents a separate unigene. Yellow and purple indicate high and low expression levels, respectively.

**Supplementary Figure S12. Phylogenetic relationships among the *Dectes texanus, Anoplophora glabripennis* and *Tribolium castaneum* sugar porters (SP).** A maximum likelihood analysis of *D. texanus* SP unigenes with a complete ORF was performed using RAxML-HPC2 Workflow on XSEDE in CIPRES Science Gateway with a Jones-Taylor-Thornton (JTT) protein substitution matrix and 1000 bootstrap replicates. Majority Rule consensus tree with a >= 50% bootstrap threshold was drawn using iTOL. Bootstrap values are represented in purple circles. Up-regulated SPs in soybean-fed larvae are highlighted with a red asterisk. Scale bar indicates branch lengths. One down-regulated SP in soybean-fed larvae is highlighted with a blue asterisk. *Drosophila melanogaster* SPs were included to resolve branches. *Dectes texanus* sequences are presented in bold.

**Supplementary Figure S13. Phylogenetic relationships among the *Dectes texanus, Anoplophora glabripennis* and *Tribolium castaneum* major facilitator superfamily (MFS).** A maximum likelihood analysis of *D. texanus* MFS unigenes with a complete ORF was performed using RAxML-HPC2 Workflow on XSEDE in CIPRES Science Gateway with a Jones-Taylor-Thornton (JTT) protein substitution matrix and 1000 bootstrap replicates. Majority Rule consensus tree with a >= 50% bootstrap threshold was drawn using iTOL. Bootstrap values are represented in purple circles. Scale bar indicates branch lengths. MFS families are shown in different colors. *Drosophila melanogaster* and *Acyrthosiphon pisum* MFS were included to resolve branches. *Dectes texanus* sequences are presented in bold. ACS= Anion:Cation Symporter, DHA1= Drug:H+ Antiporter-1 (12 Spanner), Spinster=Endosomal Spinster, ENT=Equilibrative Nucleoside Transporter, FLVCR-H=Feline Leukemia Virus Subgroup C Receptor/Heme Importer, GPH_CS=Glycoside-Pentoside-Hexuronide:Cation Symporter, MCT=Monocarboxylate Transporter, NAG-T=N-Acetylglucosamine Transporter, OCT=Organic Cation Transporter, OPA=Organophosphate:Pi Antiporter, PAT=Peptide/Acetyl-Coenzyme A/Drug Transporter, PlCUP=Plant Copper Uptake Porter, Pht=Proteobacterial Intraphagosomal Amino Acid Transporter, PCFT-HCP=Proton Coupled Folate Transporter/Heme Carrier Protein, UMF14=Unidentified Major Facilitator-14, VNT=Vesicular Neurotransmitter Transporter. Up-regulated VNT in soybean-fed larvae is highlighted with a red asterisk.
