# Supplementary material for: Comparative analyses of transcriptional responses of Dectes texanus LeConte (Coleoptera: Cerambycidae) larvae fed on three different host plants and artificial diet
Source: Sci Rep. 2021 Jun 1;11:11448. doi: 10.1038/s41598-021-90932-x (PMC8169664; doi:10.1038/s41598-021-90932-x)
Supplement: Supplementary file 3 — Supplementary Information 3. [file 41598_2021_90932_MOESM3_ESM.pdf]

Comparative analyses of transcriptional responses of *Dectes texanus* LeConte (Coleoptera: Cerambycidae) larvae fed on three different host plants and artificial diet.

Lina M. Aguirre-Rojas, Erin D. Scully, Harold N. Trick, Kun Yan Zhu, and C. Michael Smith

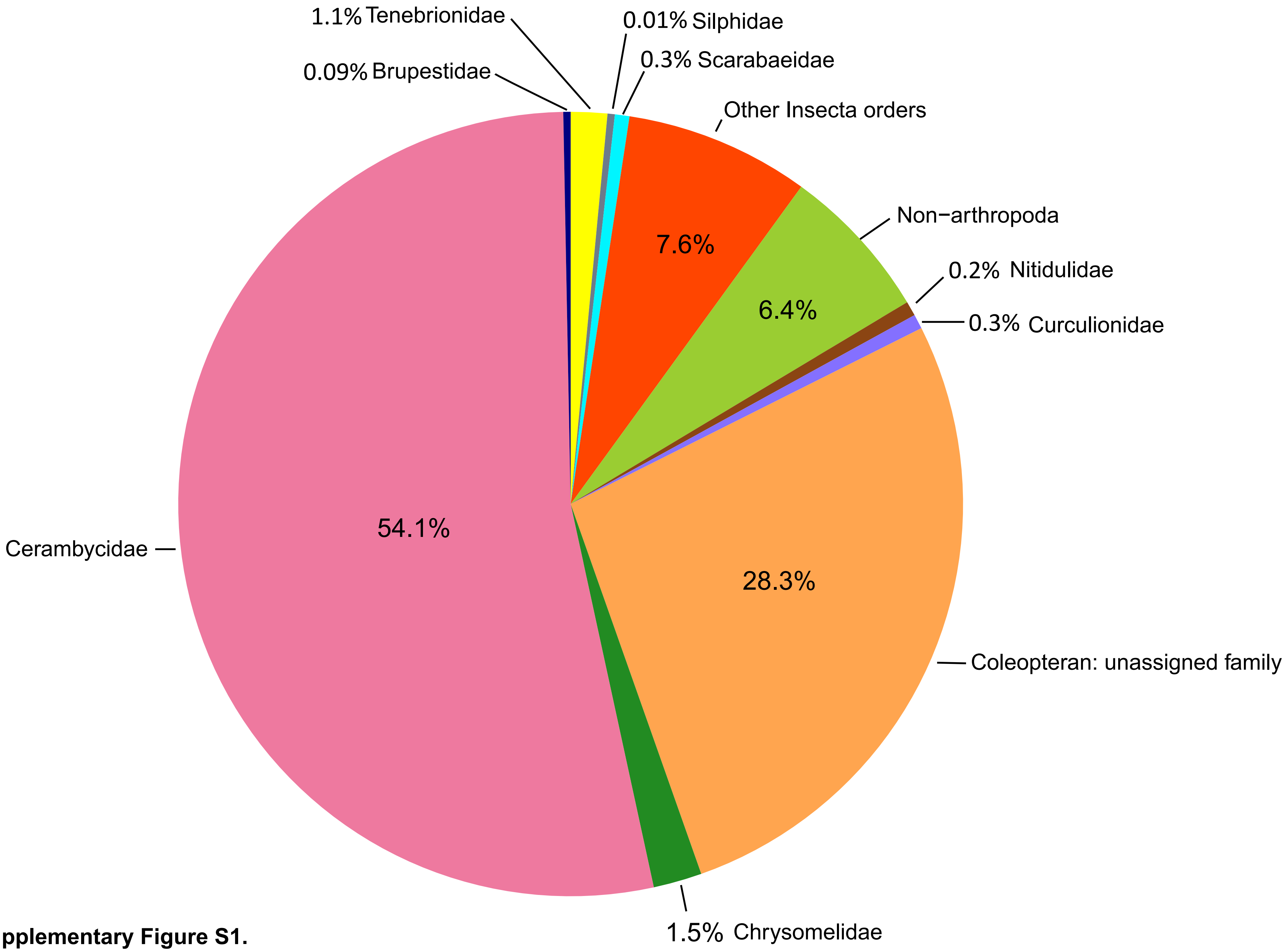

Supplementary Figure S1.

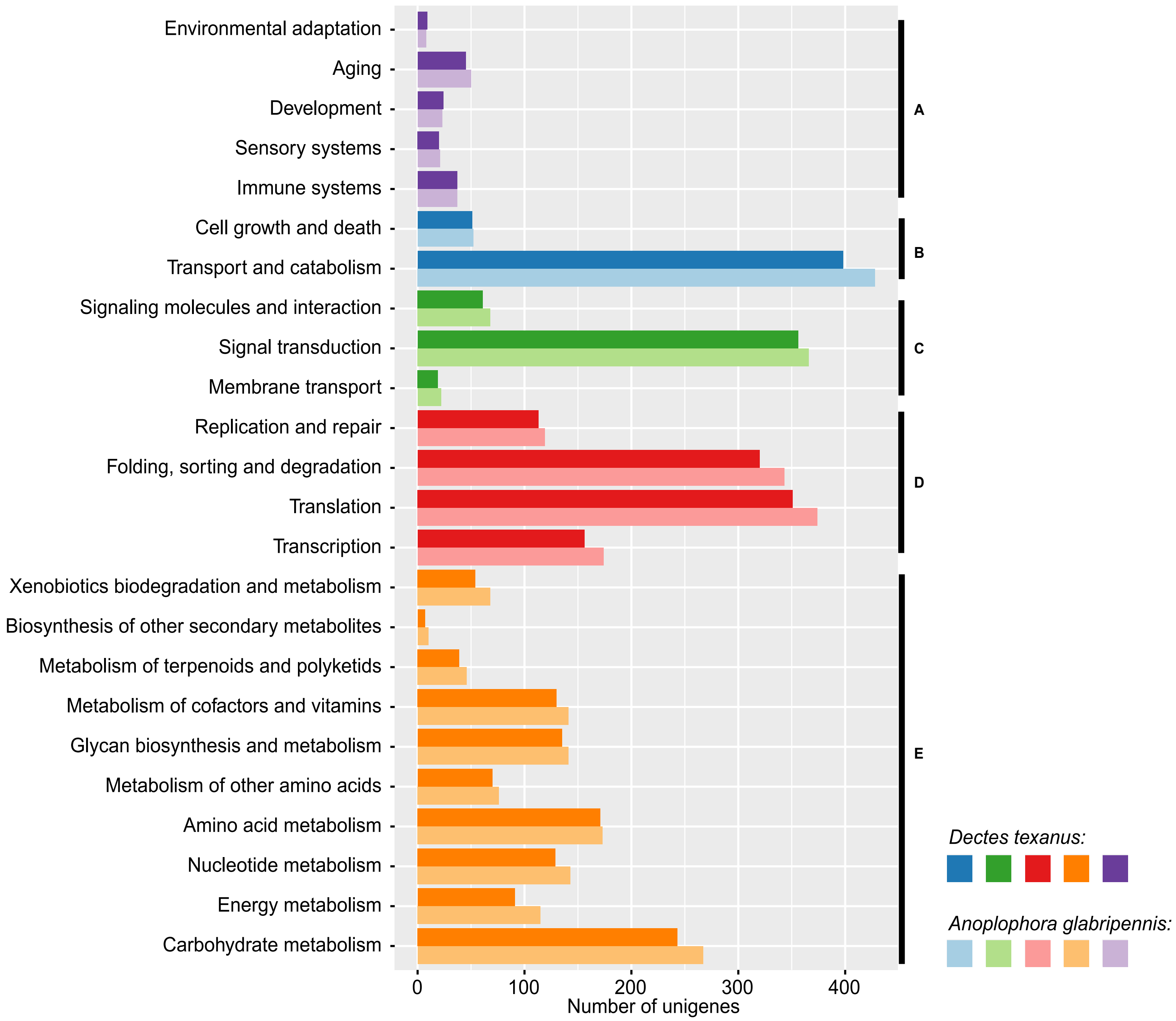

Supplementary Figure S2.

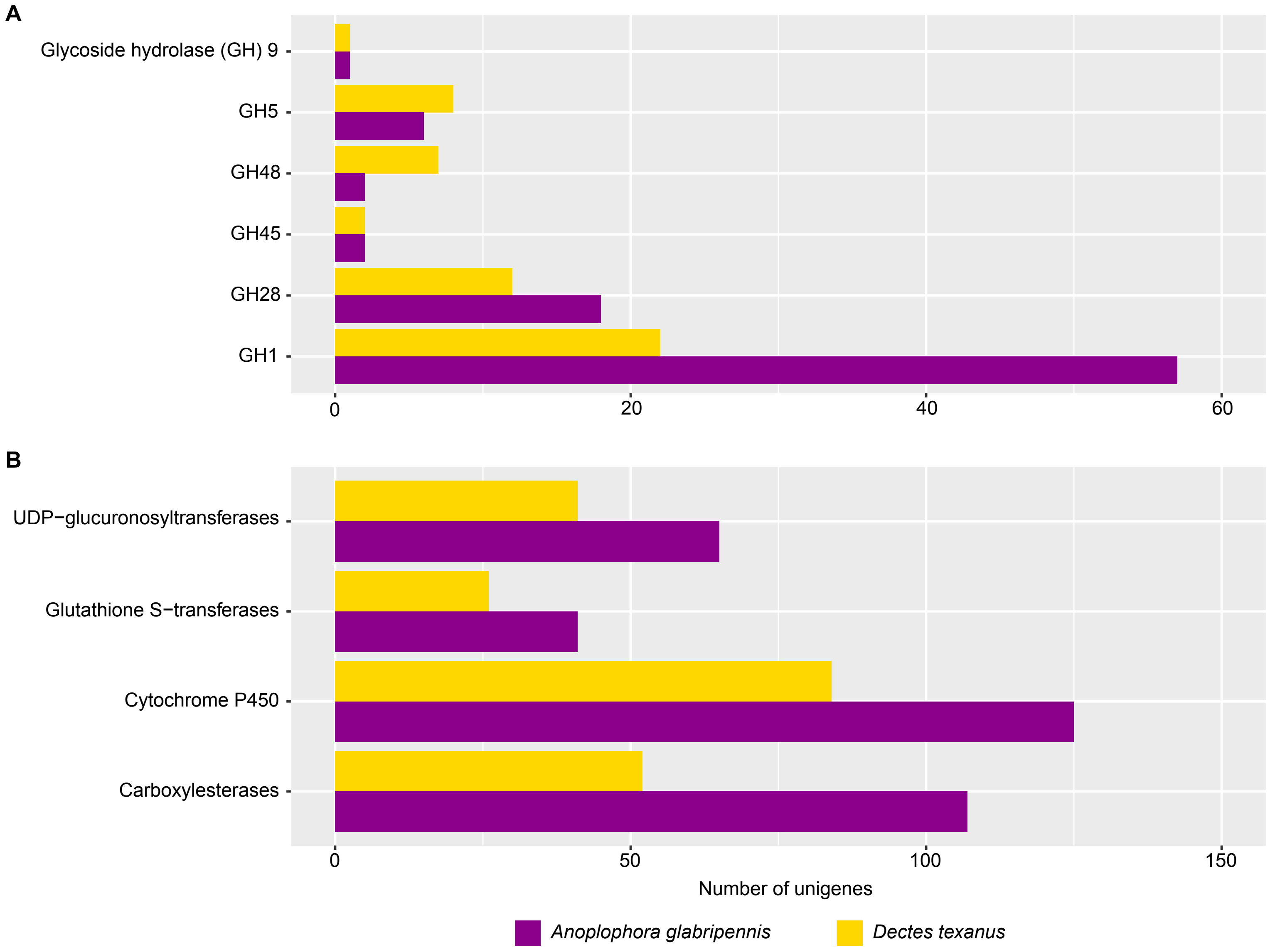

Supplementary Figure S3.

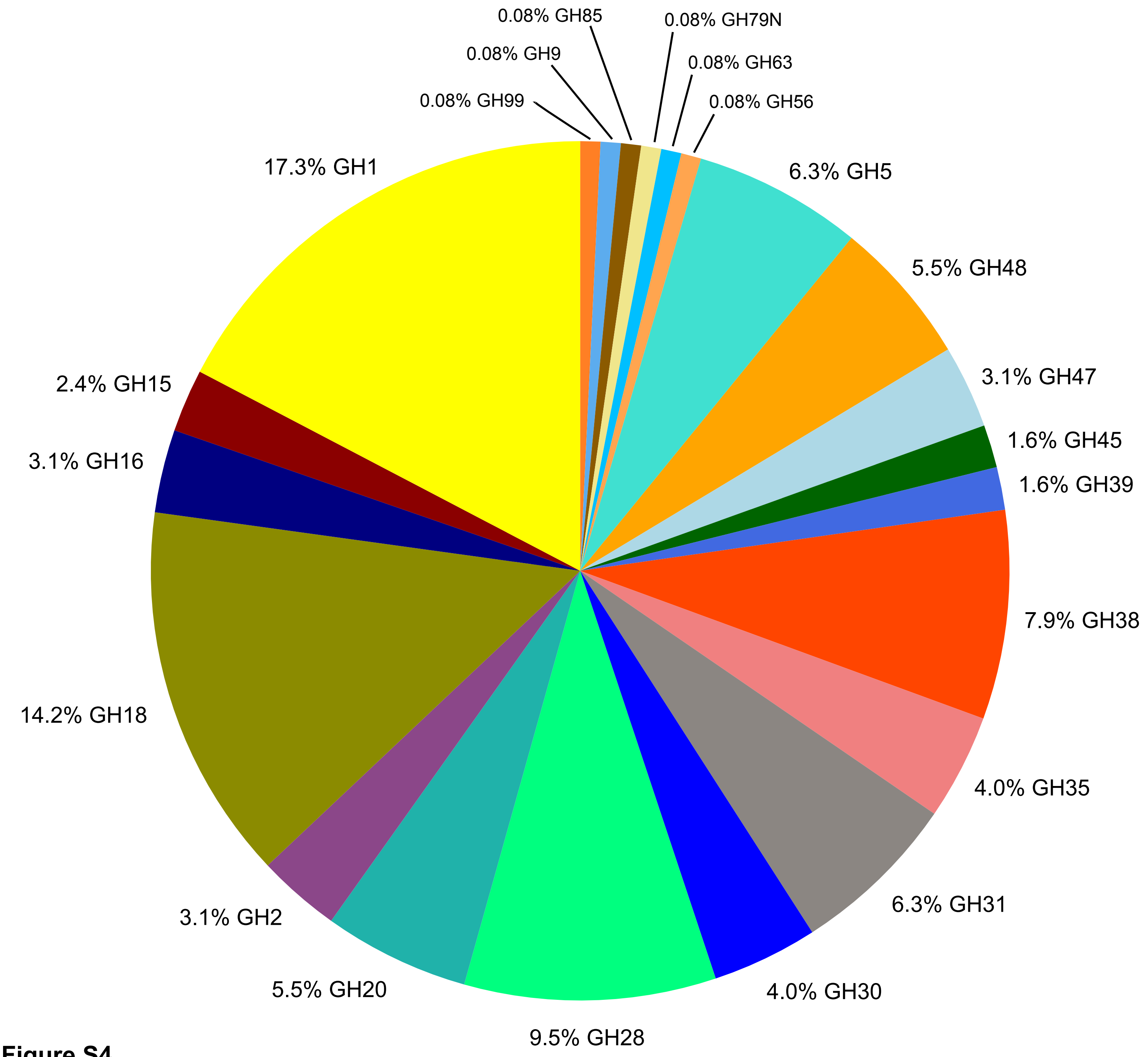

Supplementary Figure S4.

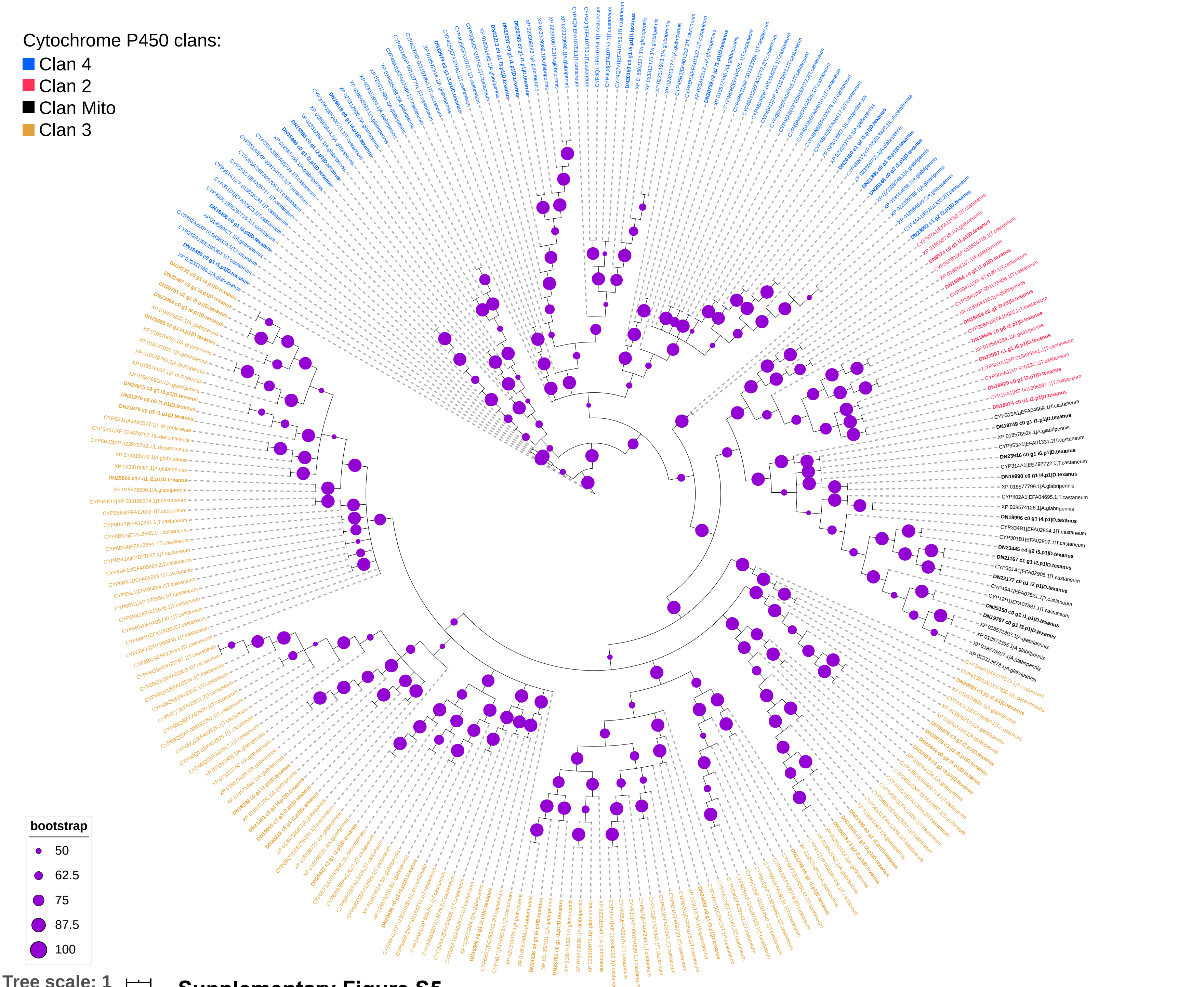

Supplementary Figure S5.

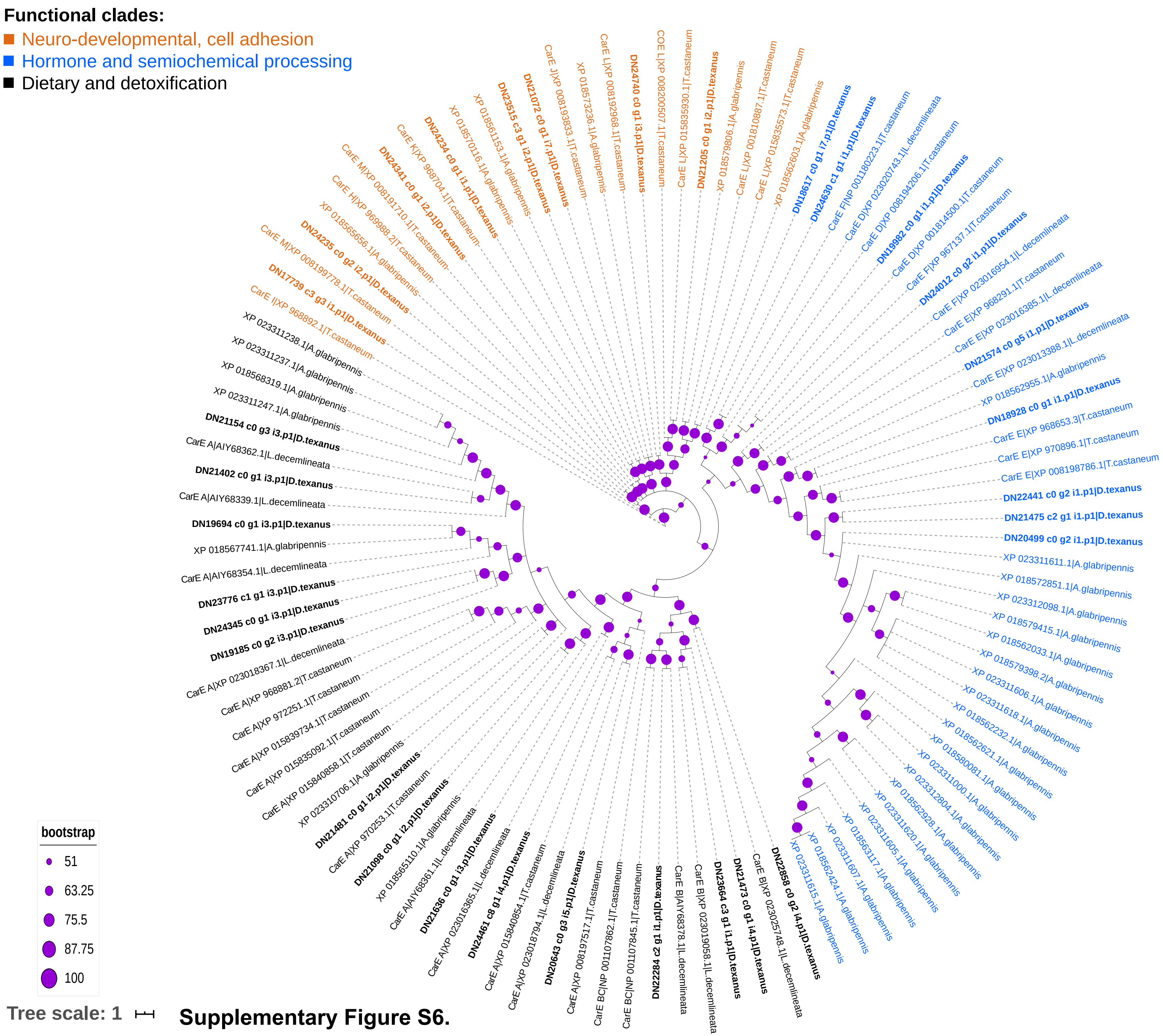

Supplementary Figure S6.

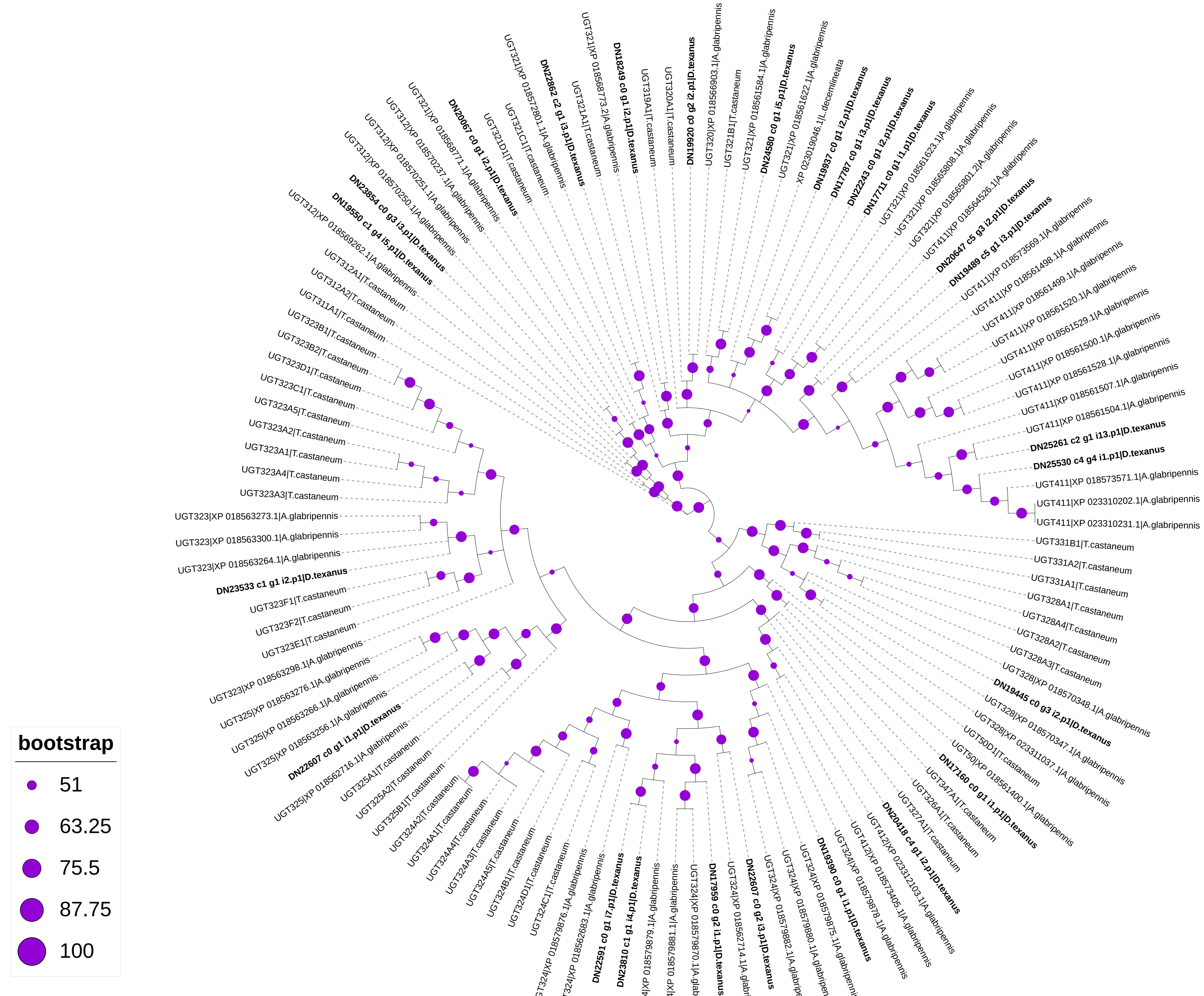

Supplementary Figure S7.

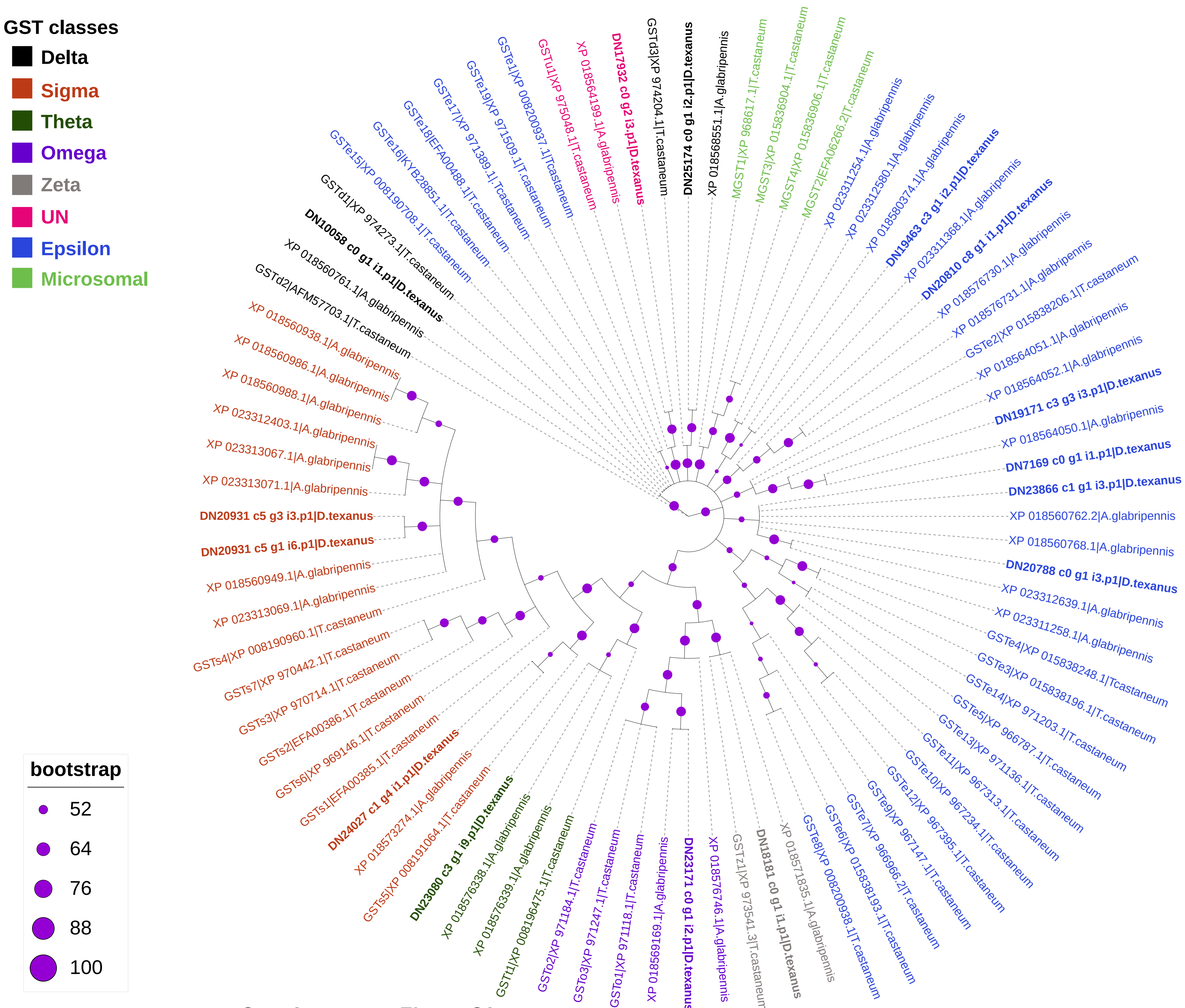

Supplementary Figure S8.

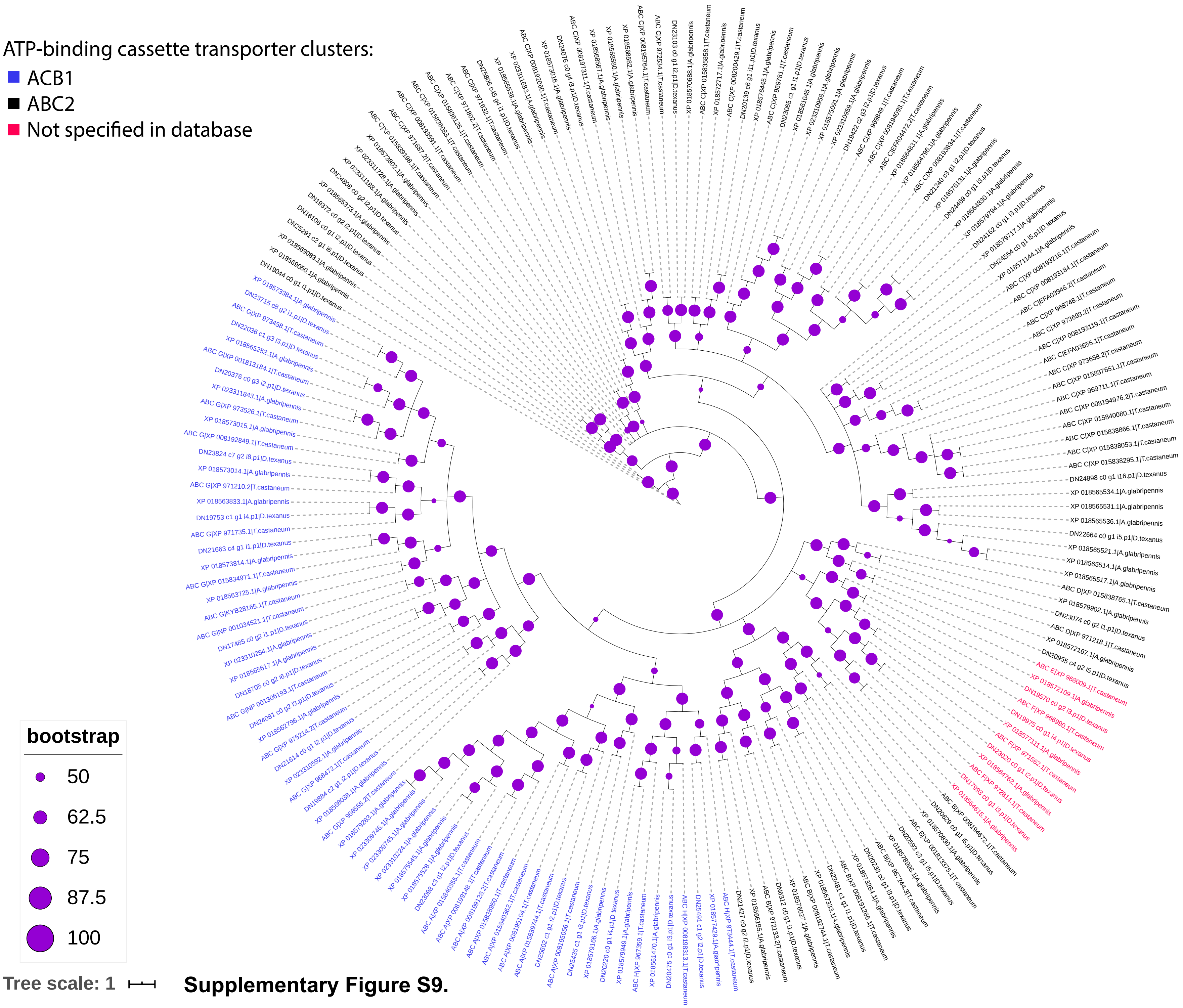

Supplementary Figure S9.

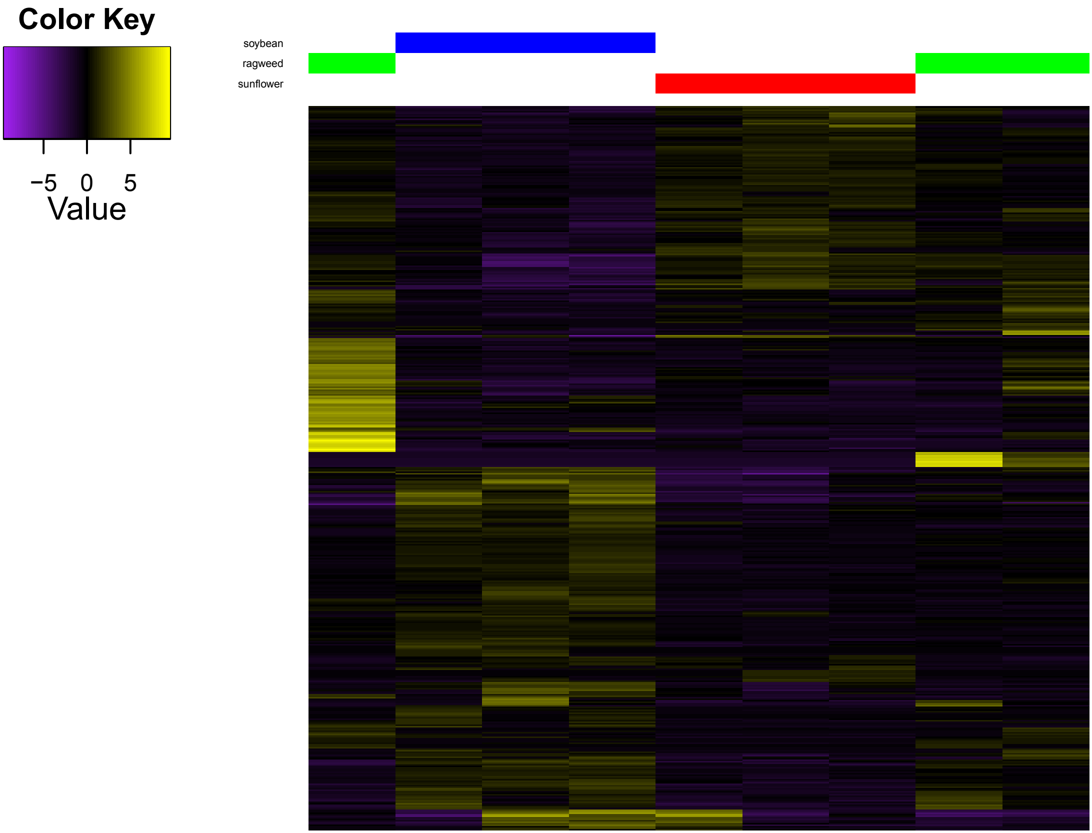

Supplementary Figure S10.

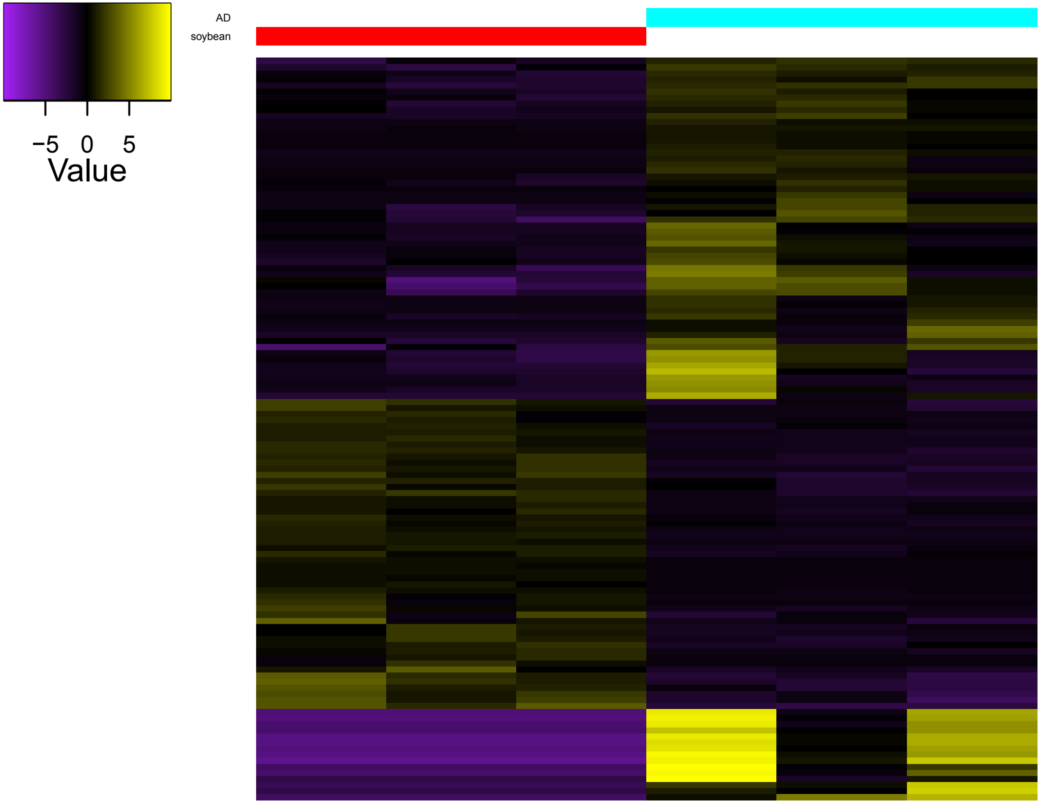

Supplementary Figure S11.

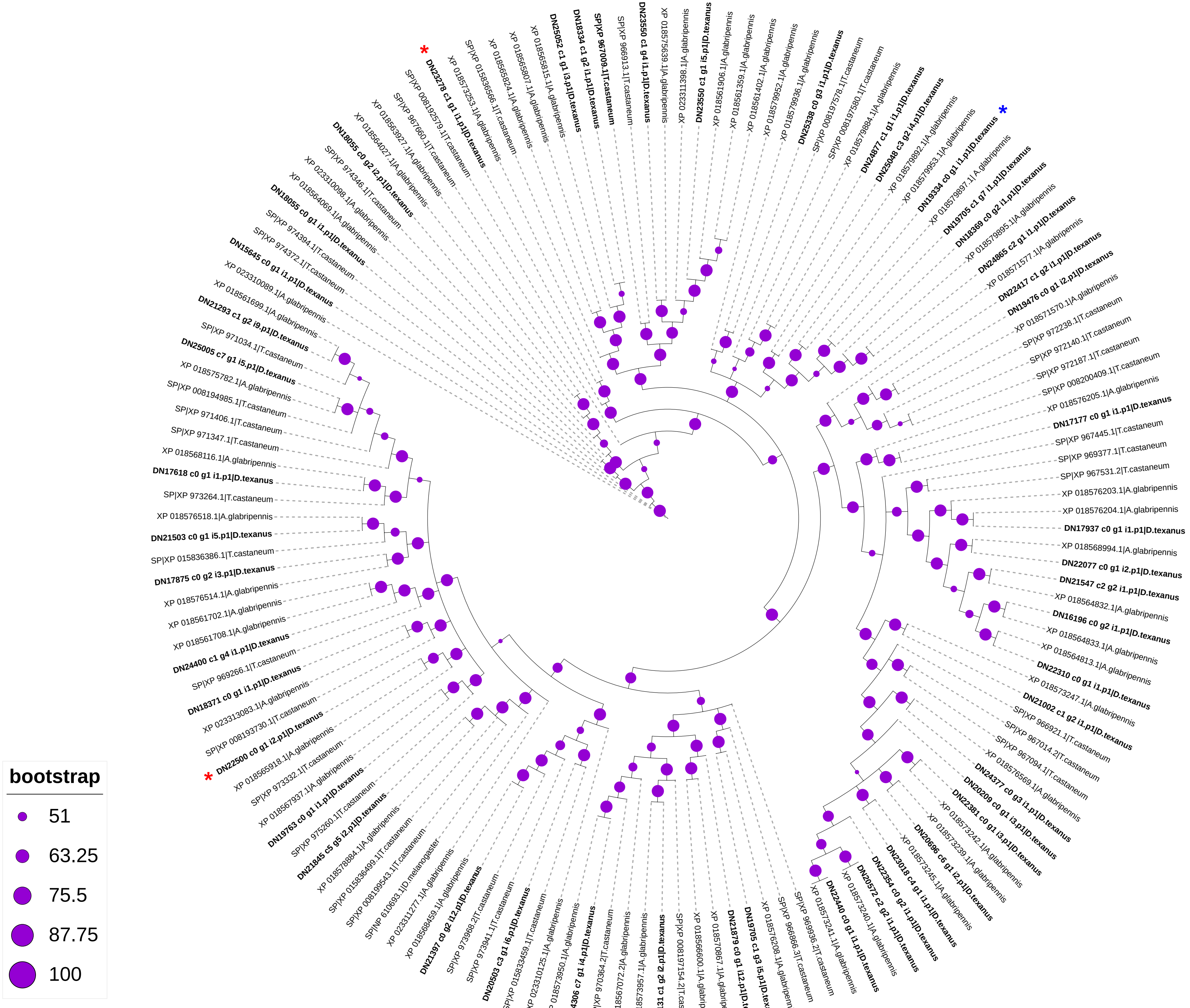

Tree scale: 1

Supplementary Figure S12.

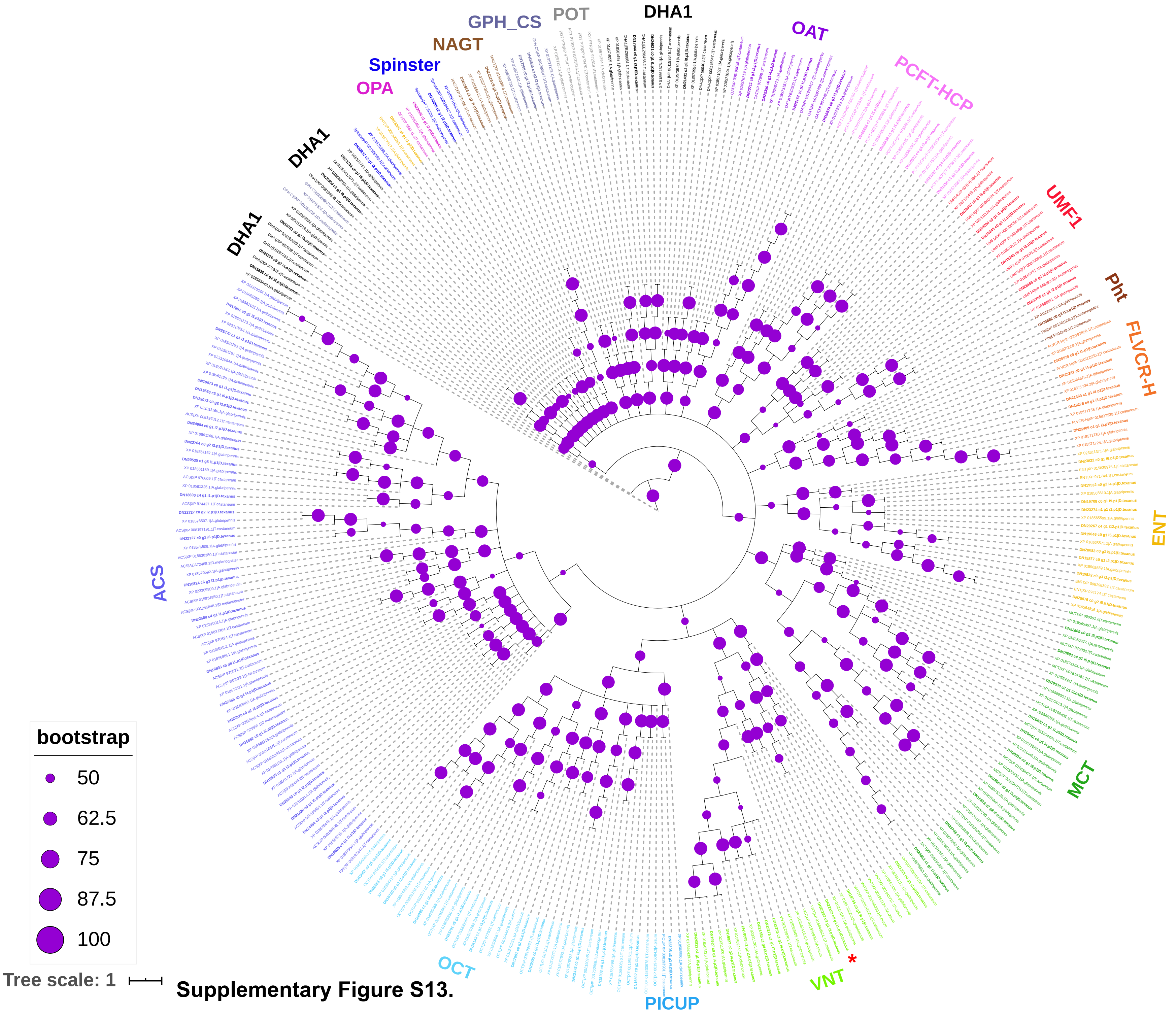

Supplementary Figure S13.
