# Supplementary material for: Comparative analyses of transcriptional responses of Dectes texanus LeConte (Coleoptera: Cerambycidae) larvae fed on three different host plants and artificial diet
Source: Sci Rep. 2021 Jun 1;11:11448. doi: 10.1038/s41598-021-90932-x (PMC8169664; doi:10.1038/s41598-021-90932-x)
Supplement: Supplementary file 4 — Supplementary Information 4. [file 41598_2021_90932_MOESM4_ESM.docx]

**Comparative analyses of transcriptional responses of *Dectes texanus* LeConte (Coleoptera: Cerambycidae) larvae fed on three different host plants and artificial diet.**

**Lina M. Aguirre-Rojas, Erin D. Scully, Harold N. Trick, Kun Yan Zhu, and C. Michael Smith**

Supplementary Table S1. Read yields from Illumina sequencing of *Dectes texanus* RNA-Seq libraries and database accessions

|  | **Raw** | **Filtered** |  | **SRA accession #** |
| --- | --- | --- | --- | --- |
| **Sample Name** | **Total Read Pairs** | **Total Read Pairs** | **Percent Retained** |  |
| Soybean-A | 29,835,970 | 29,820,640 | 99.95% | SRR13613717 |
| Soybean-B | 29,097,180 | 29,076,976 | 99.93% | SRR13613716 |
| Soybean-C | 33,825,722 | 33,804,958 | 99.94% | SRR13613713 |
| Sunflower-A | 26,460,398 | 26,446,466 | 99.95% | SRR13613712 |
| Sunflower-B | 25,787,038 | 25,770,590 | 99.94% | SRR13613711 |
| Sunflower-C | 30,650,236 | 30,635,780 | 99.95% | SRR13613710 |
| Giant ragweed-A | 30,301,950 | 30,285,848 | 99.95% | SRR13613709 |
| Giant ragweed-B | 30,046,694 | 30,027,174 | 99.94% | SRR13613708 |
| Giant ragweed-C | 31,849,712 | 31,830,198 | 99.94% | SRR13613707 |
| Artificial diet – B | 29,461,486 | 29,445,310 | 99.95% | SRR13613706 |
| Artificial diet – D | 27,837,534 | 27,822,438 | 99.95% | SRR13613715 |
| Artificial diet – E | 30,492,108 | 30,468,730 | 99.92% | SRR13613714 |
| Total Read Pairs | 355,646,028 | 355,435,108 | 99.94% |  |
| Total Gb of data | 97.3 | 97.1 | 99.79% |  |

Uppercase letters indicate different replicates. SRA = Sequence Read Archive.

Supplementary Table S2. Descriptive summary of *Dectes texanus de novo* transcriptome assembly metrics

| **Assembled Reads** | **Unfiltered** | **Filtered** |
| --- | --- | --- |
| Number of transcripts | 127,878 | 41,461 |
| Number of unigenes | 65,979 | 14,504 |
| N50 unigene length (longest transcript per unigene) (bp) | 1877 | 3,195 |
| Sum longest transcript per unigene (Mb) | 57.5 | 33.3 |
| N50 transcript length (bp) | 2,387 | 3,025 |
| Sum transcript length (Mb) | 152.1 | 97 |
| Isoforms per unigenes | 1.9 | 2.9 |
| GC % | 35.77 | 37.19 |
| N50 protein length (amino acids) | - | 585 |
| Number of transcripts with complete predicted ORF (% of total transcripts) | - | 30,601 (73.8) |
| Number of transcripts with 5’ partial ORF (% of total transcripts) | - | 3,267 (7.9) |
| Number of transcripts with 3’ partial ORF (% of total transcripts) | - | 5,199 (12.5) |
| Number of unigenes with complete predicted ORF (% of unigenes) | - | 9,975 (68.8) |
| Number of unigenes with 5’partial ORF (% of unigenes) | - | 1,588 (10.9) |
| Number of unigenes with 3’ partial ORF (% of unigenes) | - | 1,421 (9.8) |

ORF= Open reading frame

Supplementary Table S3. Summary of *Dectes texanus de novo* transcriptome annotation metrics

| **Annotation summary filtered *de novo* assembly** | **BlastP (SwissProt)** | **Pfam-A domain** | **GO** | **KEGG orthology** |
| --- | --- | --- | --- | --- |
| Number of transcripts with match (% total transcripts) | 31,866 (76.9) | 30,896 (74.5) | 20,184 (48.7) | 10,297 (24.8) |
| Number unigenes with match (% total unigenes) | 10,307 (71.1) | 10,471 (72.2) | 6,772 (46.7) | 5,362 (37.0) |

GO= Gene ontology

Supplementary Table S4. Number of KEGG orthology (KO) terms identified in the *Dectes texanus* transcriptome and beetle reference genomes. KO terms represented in each pathway were only counted once for each species.

| **KO pathway** | ***Dectes texanus* transcriptome** | ***Anoplophora glabripennis^*^*** | ***Tribolium castaneum^**^*** | ***Dendroctonus ponderosae^**^*** |
| --- | --- | --- | --- | --- |
| **Metabolism** |  |  |  |  |
| **Carbohydrate metabolism** |  |  |  |  |
| Glycolysis / Gluconeogenesis | 27 | 27 | 27 | 27 |
| Citrate cycle (TCA cycle) | 22 | 22 | 22 | 22 |
| Pentose phosphate pathway | 19 | 19 | 19 | 19 |
| Pentose and glucuronate interconversions | 12 | 12 | 12 | 13 |
| Fructose and mannose metabolism | 16 | 16 | 16 | 16 |
| Galactose metabolism | 12 | 13 | 13 | 12 |
| Ascorbate and aldarate metabolism | 6 | 6 | 6 | 6 |
| Starch and sucrose metabolism | 15 | 15 | 15 | 14 |
| Amino sugar and nucleotide sugar metabolism | 29 | 29 | 29 | 29 |
| Pyruvate metabolism | 21 | 21 | 21 | 20 |
| Glyoxylate and dicarboxylate metabolism | 21 | 21 | 21 | 22 |
| Propanoate metabolism | 20 | 20 | 20 | 20 |
| Butanoate metabolism | 11 | 12 | 12 | 11 |
| Inositol phosphate metabolism | 38 | 37 | 38 | 38 |
| **Energy metabolism** |  |  |  |  |
| Oxidative phosphorylation | 70 | 97 | 99 | 89 |
| Nitrogen metabolism | 5 | 5 | 5 | 5 |
| Sulfur metabolism | 6 | 6 | 6 | 6 |
| **Lipid metabolism** |  |  |  |  |
| Fatty acid biosynthesis | 6 | 6 | 6 | 6 |
| Fatty acid elongation | 14 | 14 | 14 | 13 |
| Fatty acid degradation | 21 | 21 | 21 | 21 |
| Synthesis and degradation of ketone bodies | 4 | 4 | 4 | 4 |
| Cutin, suberine and wax biosynthesis | 1 | 1 | 1 | 1 |
| Steroid biosynthesis | 3 | 3 | 3 | 3 |
| Glycerolipid metabolism | 21 | 21 | 22 | 21 |
| Glycerophospholipid metabolism | 41 | 41 | 41 | 41 |
| Ether lipid metabolism | 12 | 12 | 12 | 11 |
| Sphingolipid metabolism | 18 | 18 | 18 | 18 |
| Arachidonic acid metabolism | 9 | 9 | 9 | 9 |
| Linoleic acid metabolism | 3 | 3 | 3 | 3 |
| alpha-Linolenic acid metabolism | 4 | 4 | 4 | 4 |
| Biosynthesis of unsaturated fatty acids | 7 | 7 | 7 | 7 |
| **Nucleotide metabolism** |  |  |  |  |
| Purine metabolism | 94 | 98 | 102 | 101 |
| Pyrimidine metabolism | 62 | 65 | 66 | 66 |
| **Amino acid metabolism** |  |  |  |  |
| Alanine, aspartate and glutamate metabolism | 22 | 23 | 23 | 22 |
| Glycine, serine and threonine metabolism | 23 | 23 | 23 | 23 |
| Cysteine and methionine metabolism | 26 | 27 | 27 | 26 |
| Valine, leucine and isoleucine degradation | 32 | 32 | 32 | 32 |
| Lysine degradation | 30 | 30 | 30 | 31 |
| Arginine and proline metabolism | 22 | 22 | 22 | 21 |
| Histidine metabolism | 5 | 6 | 7 | 5 |
| Tyrosine metabolism | 15 | 16 | 16 | 15 |
| Phenylalanine metabolism | 8 | 8 | 8 | 8 |
| Tryptophan metabolism | 17 | 17 | 17 | 16 |
| **Metabolism of other amino acids** |  |  |  |  |
| beta-Alanine metabolism | 14 | 15 | 15 | 15 |
| Taurine and hypotaurine metabolism | 4 | 5 | 5 | 5 |
| Phosphonate and phosphinate metabolism | 3 | 3 | 3 | 3 |
| Selenocompound metabolism | 6 | 6 | 6 | 6 |
| D-Glutamine and D-glutamate metabolism | 2 | 2 | 2 | 2 |
| D-Arginine and D-ornithine metabolism | 1 | 1 | 1 | 1 |
| Glutathione metabolism | 21 | 21 | 22 | 23 |
| **Glycan biosynthesis and metabolism** |  |  |  |  |
| N-Glycan biosynthesis | 31 | 32 | 32 | 33 |
| Mucin type O-glycan biosynthesis | 2 | 2 | 2 | 2 |
| Mannose type O-glycan biosynthesis | 4 | 4 | 4 | 4 |
| Other types of O-glycan biosynthesis | 9 | 9 | 9 | 10 |
| Glycosaminoglycan biosynthesis - chondroitin sulfate / dermatan sulfate | 8 | 9 | 9 | 8 |
| Glycosaminoglycan biosynthesis - heparan sulfate / heparin | 13 | 13 | 13 | 13 |
| Glycosaminoglycan biosynthesis - keratan sulfate | 1 | 1 | 1 | 2 |
| Glycosaminoglycan degradation | 10 | 10 | 10 | 9 |
| Glycosylphosphatidylinositol (GPI)-anchor biosynthesis | 21 | 21 | 22 | 21 |
| Glycosphingolipid biosynthesis - lacto and neolacto series | 2 | 3 | 3 | 5 |
| Glycosphingolipid biosynthesis - globo and isoglobo series | 5 | 5 | 5 | 5 |
| Glycosphingolipid biosynthesis - ganglio series | 2 | 2 | 2 | 2 |
| Other glycan degradation | 11 | 11 | 11 | 10 |
| **Metabolism of cofactors and vitamins** |  |  |  |  |
| Thiamine metabolism | 5 | 5 | 6 | 6 |
| Riboflavin metabolism | 5 | 5 | 5 | 5 |
| Vitamin B6 metabolism | 4 | 4 | 4 | 4 |
| Nicotinate and nicotinamide metabolism | 9 | 9 | 9 | 9 |
| Pantothenate and CoA biosynthesis | 8 | 9 | 9 | 10 |
| Biotin metabolism | 2 | 2 | 2 | 3 |
| Lipoic acid metabolism | 3 | 3 | 3 | 3 |
| Folate biosynthesis | 19 | 19 | 19 | 19 |
| One carbon pool by folate | 9 | 10 | 10 | 12 |
| Retinol metabolism | 5 | 6 | 6 | 5 |
| Porphyrin and chlorophyll metabolism | 18 | 18 | 18 | 18 |
| Ubiquinone and other terpenoid-quinone biosynthesis | 8 | 8 | 8 | 9 |
| **Metabolism of terpenoids and polyketids** |  |  |  |  |
| Terpenoid backbone biosynthesis | 19 | 19 | 19 | 19 |
| Insect hormone biosynthesis | 13 | 13 | 13 | 13 |
| Biosynthesis of ansamycins | 1 | 1 | 1 | 1 |
| **Biosynthesis of other secondary metabolites** |  |  |  |  |
| Caffeine metabolism | 1 | 1 | 1 | 1 |
| Penicillin and cephalosporin biosynthesis | 1 | 1 | 1 | 1 |
| Monobactam biosynthesis | 1 | 1 | 1 | 1 |
| Neomycin, kanamycin and gentamicin biosynthesis | 1 | 1 | 1 | 1 |
| Prodigiosin biosynthesis | 1 | 1 | 1 | 1 |
| Aflatoxin biosynthesis | 1 | 1 | 1 | 1 |
| **Xenobiotics biodegradation and metabolism** |  |  |  |  |
| Metabolism of xenobiotics by cytochrome P450 | 6 | 6 | 6 | 6 |
| Drug metabolism - cytochrome P450 | 5 | 5 | 5 | 4 |
| Drug metabolism - other enzymes | 20 | 20 | 20 | 20 |
|  |  |  |  |  |
| ***Genetic Information Processing*** |  |  |  |  |
| **Transcription** |  |  |  |  |
| RNA polymerase | 24 | 26 | 26 | 27 |
| Basal transcription factors | 30 | 31 | 31 | 32 |
| Spliceosome | 94 | 102 | 102 | 100 |
| **Translation** |  |  |  |  |
| Ribosome | 98 | 117 | 121 | 116 |
| Aminoacyl-tRNA biosynthesis | 26 | 26 | 45 | 26 |
| RNA transport | 113 | 111 | 114 | 113 |
| mRNA surveillance pathway | 55 | 54 | 55 | 55 |
| Ribosome biogenesis in eukaryote | 59 | 62 | 64 | 63 |
| **Folding, sorting and degradation** |  |  |  |  |
| Protein export | 16 | 21 | 21 | 21 |
| Protein processing in endoplasmic reticulum | 102 | 105 | 106 | 103 |
| SNARE interactions in vesicular transport | 18 | 18 | 18 | 19 |
| Ubiquitin mediated proteolysis | 85 | 84 | 87 | 86 |
| Sulfur relay system | 6 | 6 | 7 | 6 |
| Proteasome | 36 | 37 | 37 | 35 |
| RNA degradation | 48 | 54 | 55 | 54 |
| **Replication and repair** |  |  |  |  |
| DNA replication | 31 | 32 | 32 | 32 |
| Base excision repair | 20 | 21 | 22 | 17 |
| Nucleotide excision repair | 33 | 35 | 35 | 35 |
| Mismatch repair | 18 | 18 | 18 | 18 |
| Homologous recombination | 27 | 28 | 28 | 27 |
| Non-homologous end-joining | 9 | 9 | 9 | 8 |
| Fanconi anemia pathway | 31 | 31 | 32 | 32 |
|  |  |  |  |  |
| ***Environmental Information Processing*** |  |  |  |  |
| **Membrane transport** |  |  |  |  |
| ABC transporters | 13 | 13 | 13 | 13 |
| **Signal transduction** |  |  |  |  |
| MAPK signaling pathway - fly | 78 | 78 | 80 | 79 |
| Wnt signaling pathway | 57 | 58 | 59 | 54 |
| Notch signaling pathway | 20 | 21 | 21 | 21 |
| Hedgehog signaling pathway - fly | 23 | 23 | 23 | 22 |
| TGF-beta signaling pathway | 31 | 32 | 32 | 33 |
| Hippo signaling pathway - fly | 46 | 46 | 46 | 46 |
| Hippo signaling pathway - multiple species | 15 | 15 | 15 | 15 |
| FoxO signaling pathway | 52 | 52 | 53 | 51 |
| Phosphatidylinositol signaling system | 40 | 39 | 40 | 41 |
| mTOR signaling pathway | 77 | 78 | 78 | 76 |
| **Signaling molecules and interaction** |  |  |  |  |
| Neuroactive ligand-receptor interaction | 29 | 31 | 32 | 32 |
| ECM-receptor interaction | 14 | 14 | 14 | 14 |
|  |  |  |  |  |
| ***Cellular Processes*** |  |  |  |  |
| **Transport and catabolism** |  |  |  |  |
| Endocytosis | 111 | 111 | 111 | 108 |
| Phagosome | 38 | 41 | 42 | 40 |
| Lysosome | 57 | 58 | 61 | 55 |
| Peroxisome | 49 | 48 | 49 | 43 |
| Autophagy – animal | 75 | 76 | 77 | 73 |
| Autophagy – other | 22 | 22 | 22 | 20 |
| Mitophagy – animal | 31 | 31 | 33 | 34 |
| **Cell growth and death** |  |  |  |  |
| Apoptosis – fly | 44 | 45 | 45 | 44 |
| Apoptosis - multiple species | 15 | 15 | 15 | 15 |
|  |  |  |  |  |
| ***Organismal Systems*** |  |  |  |  |
| **Immune systems** |  |  |  |  |
| Toll and Imd signaling pathway | 34 | 34 | 36 | 35 |
| **Sensory systems** |  |  |  |  |
| Phototransduction – fly | 16 | 17 | 17 | 16 |
| **Development** |  |  |  |  |
| Dorso-ventral axis formation | 21 | 21 | 21 | 21 |
| **Aging** |  |  |  |  |
| Longevity regulating pathway - multiple species | 33 | 33 | 34 | 31 |
| **Environmental adaptation** |  |  |  |  |
| Circadian rhythm – fly | 8 | 8 | 8 | 7 |

TCA= Tricarboxylic acid cycle; CoA= Coenzyme A; SNARE= Soluble N-ethylmaleimide-sensitive-factor attachment receptor; ABC= ATP-binding cassette; MAPK= Mitogen-activated protein kinase; Wnt= Wingless-Integrated; TGF= Transforming growth factor; FoxO= Forkhead box protein O; mTOR= Mammalian target of rapamycin; ECM= Extracellular matrix.

**A. glabripennis* predicted proteome from the genome assembly version GCA_000390285.2.

***T. castaneum* (GCF_000002335.3_Tcas5.2) and *D. ponderosae* (GCF_000355655.1_DendPond_male_1.0) genomes included within KAAS KEGG database^128^.

**Supplementary Table S5. Enriched Gene Ontology (GO) categories from unigenes commonly up-regulated in *Dectes texanus* larvae fed soybean compared to those fed either primary host.**

| **Category** | **Ontology** | **Description** | **# DEGs** | **FDR** |
| --- | --- | --- | --- | --- |
| GO:0005506 | MF | Iron ion binding | 5 | 0.01 |
| GO:0020037 | MF | Heme binding | 5 | 0.01 |
| GO:0046906 | MF | Tetrapyrrole binding | 5 | 0.01 |
| GO:0016705 | MF | Oxidoreductase activity, acting on paired donors, with incorporation or reduction of molecular oxygen | 5 | 0.01 |

DEG= Differentially expressed unigenes; FDR= False discovery rate; MF= Molecular function

Fold change > ± 1.5, False Discovery Rate < 0.05

**Supplementary Table S6. Enriched gene ontology categories in *Dectes texanus* larvae fed soybean compared to those fed sunflower, giant ragweed or artificial diet.**

| **Fed soybean** | **Compared to fed** | **Category** | **Ontology** | **Description** | **# DEGs** | **FDR** |
| --- | --- | --- | --- | --- | --- | --- |
| Up regulated | Sunflower | GO:0055085 | BP | Transmembrane transport | 14 | 0.03 |
|  |  | GO:0055114 | BP | Oxidation-reduction process | 18 | < 0.01 |
|  |  | GO:0046914 | MF | Transition metal ion binding | 18 | < 0.01 |
|  |  | GO:0016491 | MF | Oxidoreductase activity | 17 | < 0.01 |
|  |  | GO:0022857 | MF | Transmembrane transporter activity | 16 | 0.01 |
|  |  | GO:0005215 | MF | Transporter activity | 16 | 0.01 |
|  |  | GO:0005506 | MF | Iron ion binding | 16 | < 0.01 |
|  |  | GO:0020037 | MF | Heme binding | 15 | < 0.01 |
|  |  | GO:0046906 | MF | Tetrapyrrole binding | 15 | < 0.01 |
|  |  | GO:0016705 | MF | Oxidoreductase activity, acting on paired donors, with incorporation or reduction of molecular oxygen | 15 | < 0.01 |
|  |  | GO:0044699 | N/A | Uncharacterized | 38 | 0.01 |
| Up regulated | Giant ragweed | GO:0006030 | BP | Chitin metabolic process | 7 | < 0.01 |
|  |  | GO:0006040 | BP | Amino sugar metabolic process | 7 | < 0.01 |
|  |  | GO:1901071 | BP | Glucosamine-containing compound metabolic process | 7 | < 0.01 |
|  |  | GO:0006022 | BP | Aminoglycan metabolic process | 7 | < 0.01 |
|  |  | GO:1901135 | BP | Carbohydrate derivative metabolic process | 7 | < 0.01 |
|  |  | GO:1901564 | BP | Organonitrogen compound metabolic process | 7 | 0.02 |
|  |  | GO:0020037 | MF | Heme binding | 5 | 0.04 |
|  |  | GO:0046906 | MF | Tetrapyrrole binding | 5 | 0.04 |
|  |  | GO:0016705 | MF | Oxidoreductase activity, acting on paired donors, with incorporation or reduction of molecular oxygen | 5 | 0.04 |
|  |  | GO:0005506 | MF | Iron ion binding | 5 | < 0.05 |
|  |  | GO:0008061 | MF | Chitin binding | 7 | < 0.01 |
|  |  | GO:0005576 | CC | Extracellular region | 8 | < 0.01 |
| Up regulated | Artificial diet | N/A | N/A | N/A | N/A | N/A |
| Down regulated | Sunflower | GO:0003810 | MF | Protein-glutamine gamma-glutamyltransferase activity | 3 | 0.01 |
|  |  | GO:0016755 | MF | Transferase activity, transferring amino-acyl groups | 3 | 0.04 |
|  |  | GO:0018149 | BP | Peptide cross-linking | 3 | 0.01 |
| Down regulated | Giant ragweed | GO:0042302 | MF | Structural constituent of cuticle | 17 | < 0.01 |
|  |  | GO:0005198 | MF | Structural molecule activity | 18 | < 0.01 |
| Down regulated | Artificial diet | GO:0003968 | MF | RNA-directed 5'-3' RNA polymerase activity | 4 | < 0.01 |
|  |  | GO:0042302 | MF | Structural constituent of cuticle | 10 | < 0.01 |
|  |  | GO:0003724 | MF | RNA helicase activity | 2 | 0.03 |

BP= Biological process; MF= Molecular function; CC= Cellular component; DEG= Differentially expressed unigenes; FDR= False discovery rate.

Fold change > ± 1.5, False Discovery Rate < 0.05

**Supplementary Table S7. Enriched Gene Ontology (GO) categories from the cluster of up-regulated unigenes in *Dectes texanus* larvae fed soybean compared to those fed sunflower and giant ragweed by K-means analysis.**

| **Category** | **Ontology** | **Description** | **# DEGs** | **FDR** |
| --- | --- | --- | --- | --- |
| GO:0004650 | MF | Polygalacturonase activity | 3 | 0.02 |
| GO:0005506 | MF | Iron ion binding | 4 | 0.04 |
| GO:0020037 | MF | Heme binding | 4 | 0.04 |
| GO:0046906 | MF | Tetrapyrrole binding | 4 | 0.04 |
| GO:0016705 | MF | Oxidoreductase activity, acting on paired donors, with incorporation or reduction of molecular oxygen | 4 | 0.04 |

DEG= Differentially expressed unigenes; FDR= False discovery rate; MF= Molecular function

Fold change > ± 1.5, False Discovery Rate < 0.05

**Supplementary Table S8. Family classification of *Dectes texanus*, *Anoplophora glabripennis,* and *Tribolium castaneum* major facilitator superfamily (MFS) transporters against the Transporter Classification Database.**

Due to its size, this table has been moved to the supplementary excel file entitled Supplementary Table S8 and S9.

**Supplementary Table S9. Family classification of *Dectes texanus* and *Anoplophora glabripennis* ATP-binding cassette (ABC) transporters against the Transporter Classification Database.**

Due to its size, this table has been moved to the supplementary excel file entitled Supplementary Table S8 and S9.
